# Crosstalk between E-Cadherin/β-Catenin and NF-κB Signaling Pathways: The Regulation of Host-Pathogen Interaction during Leptospirosis

**DOI:** 10.3390/ijms222313132

**Published:** 2021-12-04

**Authors:** Shen-Hsing Hsu, Li-Fang Chou, Chung-Hung Hong, Ming-Yang Chang, Chung-Ying Tsai, Ya-Chung Tian, Huang-Yu Yang, Chih-Wei Yang

**Affiliations:** Kidney Research Center, Department of Nephrology, Chang Gung Memorial Hospital, College of Medicine, Chang Gung University, 5 Fu-Shing St., Taoyuan 33333, Taiwan; d928208@gmail.com (L.-F.C.); hongchunghung@gmail.com (C.-H.H.); mingyc@adm.cgmh.org.tw (M.-Y.C.); cytsai0616@cgmh.org.tw (C.-Y.T.); dryctian@cgmh.org.tw (Y.-C.T.); hyyang01@gmail.com (H.-Y.Y.)

**Keywords:** biomarker, β-catenin, E-cadherin, MMP7, NF-κB, NGAL, leptospirosis, leucine-rich repeat

## Abstract

Approximately 1 million cases of leptospirosis, an emerging infectious zoonotic disease, are reported each year. Pathogenic *Leptospira* species express leucine-rich repeat (LRR) proteins that are rarely expressed in non-pathogenic *Leptospira* species. The LRR domain-containing protein family is vital for the virulence of pathogenic *Leptospira* species. In this study, the biological mechanisms of an essential LRR domain protein from pathogenic *Leptospira* were examined. The effects of *Leptospira* and recombinant LRR20 (rLRR20) on the expression levels of factors involved in signal transduction were examined using microarray, quantitative real-time polymerase chain reaction, and western blotting. The secreted biomarkers were measured using an enzyme-linked immunosorbent assay. rLRR20 colocalized with E-cadherin on the cell surface and activated the downstream transcription factor β-catenin, which subsequently promoted the expression of MMP7, a kidney injury biomarker. Additionally, MMP7 inhibitors were used to demonstrate that the secreted MMP7 degrades surface E-cadherin. This feedback inhibition mechanism downregulated surface E-cadherin expression and inhibited the colonization of Leptospira. The degradation of surface E-cadherin activated the NF-κB signal transduction pathway. Leptospirosis-associated acute kidney injury is associated with the secretion of NGAL, a downstream upregulated biomarker of the NF-κB signal transduction pathway. A working model was proposed to illustrate the crosstalk between E-cadherin/β-catenin and NF-κB signal transduction pathways during *Leptospira* infection. Thus, rLRR20 of Leptospira induces kidney injury in host cells and inhibits the adhesion and invasion of *Leptospira* through the upregulation of MMP7 and NGAL.

## 1. Introduction

In the last few decades, several biomarkers have been reported for acute and chronic kidney diseases [1,2]. The mechanisms of several biomarkers, including kidney injury molecule-1 (Kim-1) [3,4], matrix metalloproteinase 7 (MMP7) [5], neutrophil gelatinase-associated lipocalin (NGAL) [6], and tissue inhibitor of metalloproteinases-2/insulin-like growth factor-binding protein 7 (TIMP2/IGFBP7), in the pathogenesis of acute and chronic kidney diseases have been elucidated [7,8]. Previous studies have reported that MMP7 and NGAL are also associated with the pathogenesis of leptospirosis [9,10]. MMP7, a zinc and calcium-dependent endopeptidase, degrades various extracellular matrix components, including collagen IV, laminin, gelatin, and fibronectin [11]. Structurally, MMP7 is a secretory protein whose structure contains the following three domains: an N-terminal signal peptide domain, a pro-domain, and a catalytic domain at the C-terminal [7,11]. MMP7 is synthesized in an inactive form, and the removal of the pro-domain activates the enzyme [5,12]. Previous studies have reported that MMP7 regulates various biological processes, such as cell–cell interaction, cell migration, cell proliferation, and cell polarization through the degradation of E-cadherin on the cell surface [13,14]. Recently, MMP7 was identified as a key biomarker for renal injury and fibrosis [5,7,12]. NGAL, a novel biomarker for acute kidney injury (AKI), is involved in the pathogenesis of various diseases, such as sepsis, cardiac disease, and cardiorenal syndrome [15]. One study reported that the urine and plasma NGAL levels are highly correlated with leptospirosis-associated AKI [9]. The authors reported the role of NGAL in leptospirosis and demonstrated the correlation between Leptospira outer membrane components and cell surface receptors. However, the roles of MMP7 and NGAL in leptospirosis pathogenesis are unclear. In this study, a potential mechanism involved in protecting the host cells against Leptospira infection was elucidated.

*Leptospira* is reported to interact with various cell surface receptors, including E-cadherin (CDH1), P-cadherin (CDH3), and VE-cadherin (CDH5) [16]. One of the LRR domain-containing proteins (LIC10831) from pathogenic *Leptospira* was reported to interact with the human E- and VE-cadherins [17]. E-cadherin is a type I transmembrane receptor that functions as a cell adhesion molecule [18]. The structure of E-cadherin contains five extracellular domains (EC1–5), a transmembrane domain, and an intracellular domain. The extracellular domains, to which Ca^2+^ ions bind, interact with other E-cadherin molecules. The intracellular domain of E-cadherin is connected with several essential signal transduction components, including α-catenin (CTNNA1), β-catenin (CTNNB1), and p120 (CTNND1) [19]. In animal cells, catenin molecules interact and form a complex with cadherins. CTNNA1 and CTNNB1 were the first two catenin molecules to be identified. CTNNA1, which can bind to CTNNB1 or actin, can interact with some cadherin molecules in the cytoplasm. As CTNNB1 expression is upregulated in some cancer cells, it is considered to be associated with carcinogenesis [20]. E-cadherin forms a dynamic complex with these signal transduction components and regulates several signal transduction pathways, such as the NF-κB, Wnt/β-catenin, PI3K/Akt, and Rho GTPase signal pathways [21]. Previous studies demonstrated that *Leptospira* infection induced a decrease of E-cadherin from the plasma membrane and the relationship between E-cadherin and *Leptospira* needs further investigation [22,23].

Previously, we demonstrated that LSS_11580 (rLRR20) was located at the outer membrane in pathogenic *Leptospira* and interacted with the EC1 domain of E-cadherin through charge–charge interactions [24]. Three essential residues (Asp^56^, Glu^59^, and Glu^123^) were proposed to interact with the EC1 domain of E-cadherin [24]. In this study, we further demonstrated that rLRR20 colocalized with E-cadherin on the cell surface and upregulated the levels of activated β-catenin, which is the downstream signal component of E-cadherin. Activated β-catenin translocated into the nucleus and consequently promoted MMP7 expression. Subsequently, MMP7 was secreted, which led to the degradation of E-cadherin on the cell surface. Meanwhile, the downregulation of surface E-cadherin also activated the NF-κB pathway through several phosphorylation mechanisms. Activated NF-κB translocated into the nucleus and consequently promoted NGAL expression [25]. Thus, rLRR20 from pathogenic *Leptospira* is a novel human E-cadherin-binding protein that promotes the expression of MMP7 and NGAL. Furthermore, rLRR20 promoted the MMP7-mediated degradation of surface E-cadherin, which led to the activation of the NF-κB pathway and the stimulation of NGAL expression.

## 2. Results

### 2.1. Microarray Analysis

**Figure 1:**
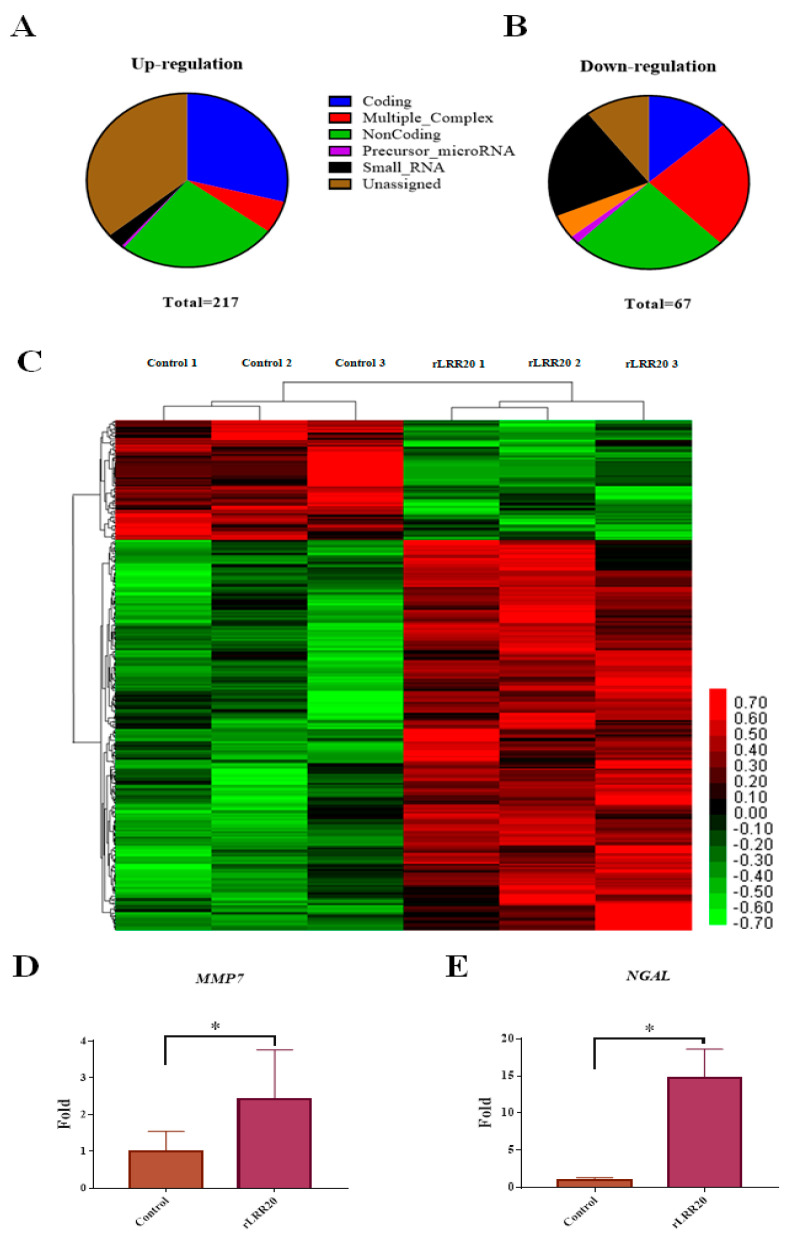
Overall representation of the effect of rLRR20 on the HK2s transcriptome profiles. The differential expression of coding and non-coding RNAs in HK2s. HK2s were treated with phosphate-buffered saline (control) or 10 μM LRR20 for 8 h. Graphs show upregulated (**A**) and downregulated (**B**) genes between the rLRR20-treated and control groups. Six groups of genes are color-coded as follows: blue, coding genes; red, multigene complex; green, non-coding; purple, precursor microRNA; black, small RNA; brown, unassigned. (**C**) Heatmap of the genes regulated by rLRR20 in HK2s. The genes with fold change >2 and *p*-value < 0.05 were analyzed. Upregulation and downregulation are indicated in red and green colors, respectively. Treatment with rLRR20 upregulated the levels of *MMP7* (**D**) and *NGAL* (**E**). * *p* < 0.05.

To examine the pathogenic mechanisms of rLRR20, the gene expression profiles of control and rLRR20-treated HK2s were comparatively analyzed using Clariom D Human array chips [26]. The purified rLRR20 protein (10 μM) was used to incubate the human kidney 2 cells (HK2s) for 4 h (Figure S1). In total, 217 genes were upregulated (>2-fold), and 67 genes were downregulated (<0.5-fold) between the control and rLRR20-treated cells (Figure 1A,B). Interestingly, two kidney injury-associated genes (*MMP7* and *NGAL*) were upregulated, indicating that these genes were involved in the rLRR20-mediated effects on kidney cells (Figure 1C–E and Table S1). The expression levels of *MMP7* and *NGAL* in the rLRR20-treated group were significantly higher than those in the control group (Figure 1D,E). The rLRR20-mediated regulation of *MMP7* and *NGAL* was further investigated to verify the correlation between leptospirosis and kidney injury. The gene IDs of the heatmap are listed in Table S1.

### 2.2. rLRR20 Interacts with E-Cadherin on the Cell Surface and Mediates Its Degradation

**Figure 2:**
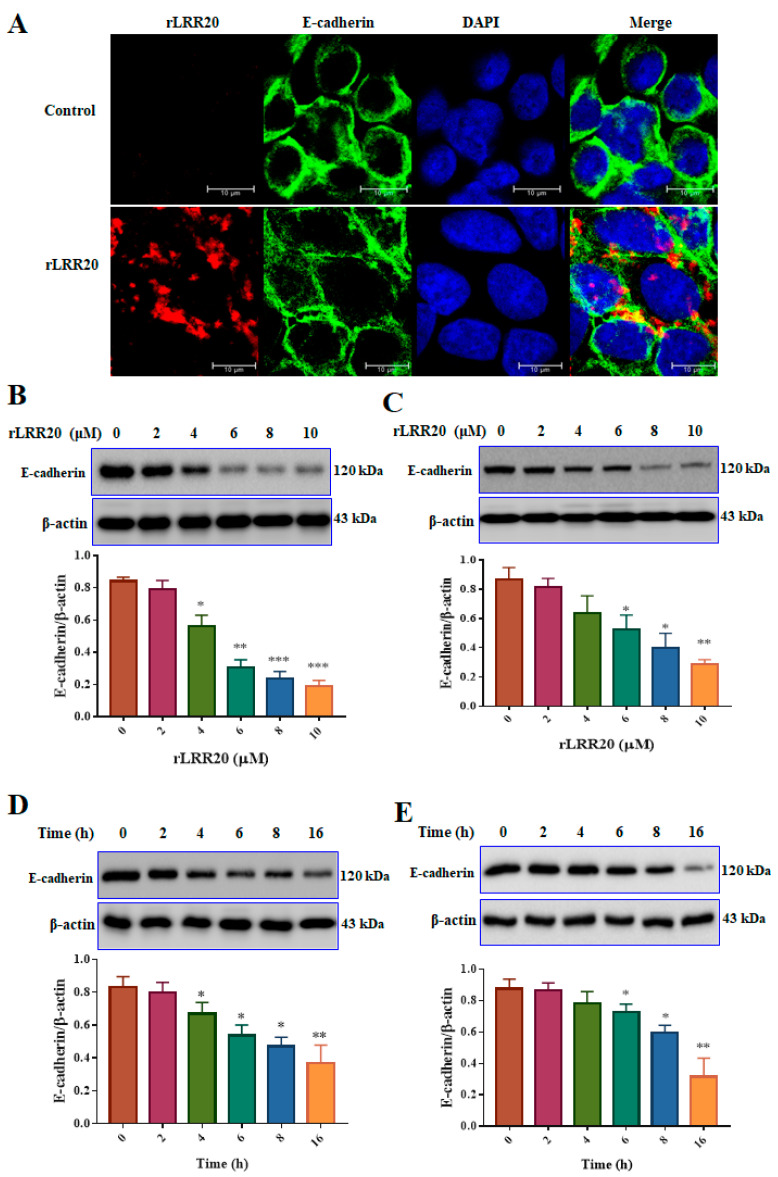
rLRR20 colocalized with E-cadherin on the cell surface and downregulated its expression. (**A**) rLRR20 colocalized with E-cadherin on the cell surface. Blue, nucleus; green, E-cadherin; red, rLRR20; yellow, colocalization of rLRR20 and E-cadherin. Effect of treatment with different doses (0, 2, 4, 6, 8, and 10 μM) of rLRR20 for 8 h on the E-cadherin protein levels in HK2s (**B**) hRPTECs (**C**). Effect of treatment with 10 μM rLRR20 for 0, 2, 4, 6, 8, or 16 h on the E-cadherin protein levels in HK2s (**D**) and hRPTECs (**E**). β-actin was used the internal control. *** *p* < 0.001; ** *p* < 0.01; * *p* < 0.05.

E-cadherin is the major cell surface receptor involved in intracellular communications. To examine the correlation between E-cadherin and rLRR20, HK2s cultured to 70% confluence were cultured under serum-free conditions for 16 h. Confocal microscopy analysis revealed that rLRR20 interacted and colocalized with E-cadherin after 2 h of incubation (Figure 2A). Additionally, the effect of treatment with different concentrations (0, 2, 4, 6, 8, and 10 μM) of rLRR20 for 8 h on the expression of E-cadherin in HK2s and human renal proximal tubular epithelial cells (hRPTECs) was examined using qRT-PCR and western blotting. Treatment with ≥4 and ≥6 μM rLRR20 significantly downregulated the E-cadherin protein levels in HK2s and hRPTECs, respectively (Figure 2B,C). Additionally, the E-cadherin protein levels in HK2s and hRPTECs treated with 10 μM rLRR20 for 0, 2, 4, 6, 8, or 16 h were examined using western blotting. Treatment with rLRR20 for ≥4 and ≥6 h significantly downregulated the E-cadherin protein levels in HK2s and hRPTECs, respectively (Figure 2D,E). The *E-cadherin* mRNA levels were non-significantly downregulated upon treatment with 8 and 10 μM rLRR20 in both HK2s and hRPTECs (Figure S2A,B). Additionally, the *E-cadherin* mRNA levels in the rLRR20-treated HK2s and hRPTECs were similar to those in the control HK2s and hRPTECs (Figure S2C,D), respectively, after treatment with rLRR20 for different durations. These findings indicated that rLRR20 regulated the expression of E-cadherin at the protein level but not at the mRNA level.

**Figure 3:**
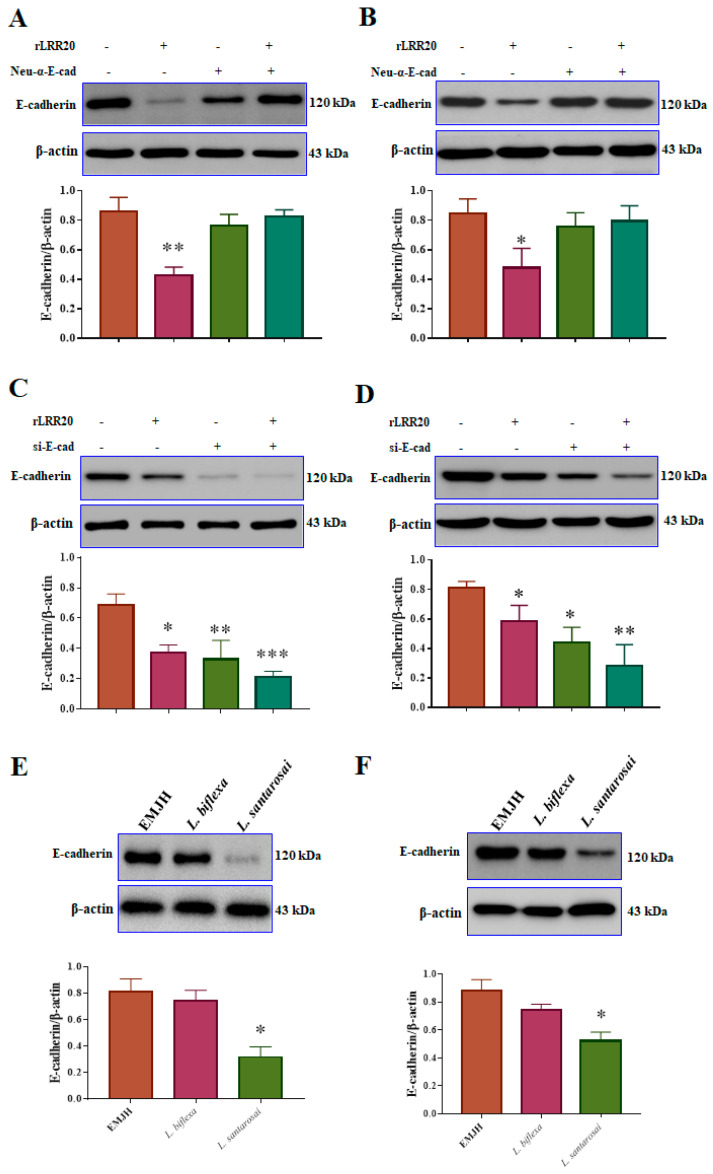
rLRR20 and *Leptospira* infection downregulated E-cadherin expression. Neutralizing anti-E-cadherin antibody (Neu-α-E-cad) and short interfering RNA against *E-cadherin* (si-E-cad) were used to verify the regulatory effects of rLRR20 on E-cadherin expression in HK2s and hRPTECs. Effect of α-E-cad, which inhibits the interaction between rLRR20 and E-cadherin, on the rLRR20-mediated degradation of E-cadherin in HK2s (**A**) and hRPTECs (**B**). Effect of si-E-cad on the rLRR20-mediated degradation of E-cadherin in HK2s (**C**) and hRPTECs (**D**). Effect of pathogenic (*L. santarosai*) and non-pathogenic (*L. biflexa*) *Leptospira* species on the E-cadherin levels in HK2s (**E**) and hRPTECs (**F**). β-actin was used as the internal control. EMJH, Ellinghausen–McCullough–Johnson–Harris medium. *** *p* < 0.001; ** *p* < 0.01; * *p* < 0.05.

To verify the rLRR20-mediated regulation of E-cadherin expression, the interaction between rLRR20 and E-cadherin was inhibited using the neutralizing anti-E-cadherin antibody (Neu-α-E-cad; #16-3249-82, Invitrogen). The effect of the Neu-α-E-cad on the E-cadherin protein levels was examined using western blotting. The HK2s and hRPTECs were incubated with Neu-α-E-cad for 1 h before adding 10 μM rLRR20 for 8 h. The E-cadherin protein levels in the rLRR20-treated HK2s and hRPTECs were significantly lower than those in the PBS-treated HK2s (Figure 3A,B). Meanwhile, the E-cadherin protein levels were similar between the rLRR20-treated and PBS-treated HK2s upon pretreatment with Neu-α-E-cad for 1 h before treatment with rLRR20 (Figure 3A,B). These findings indicated that pretreatment with Neu-α-E-cad efficiently inhibited the interaction between rLRR20 and E-cadherin. To examine the regulatory effects of rLRR20 on the *E-cadherin* RNA level, the HK2s were incubated with short interfering RNA against *E-cadherin* (si-E-cad) for 16 h (Table 1). At a concentration of 5 nM, si-E-cad significantly downregulated the expression of *E-cadherin* (Figure S2E). Therefore, 5 nM si-E-cad was used to silence *E-cadherin* mRNA. The si-E-cad-treated HK2s and hRPTECs were then incubated with 10 μM rLRR20 for 8 h, and the E-cadherin protein levels were analyzed using western blotting. The E-cadherin protein levels in the rLRR20-treated HK2s and hRPTECs were significantly downregulated when compared with those in the PBS-treated HK2s (Figure 3C,D). Compared with the PBS-treated HK2s and hRPTECs, the E-cadherin protein levels were significantly downregulated in the si-E-cad/PBS-treated HK2s (Figure 3C,D). The E-cadherin protein levels in the si-E-cad/rLRR20-treated HK2s and hRPTECs were significantly downregulated compared with those in the PBS-treated HK2s (Figure 3C,D). These findings indicated that pretreatment with the si-E-cad potentiated the rLRR20-mediated downregulation of E-cadherin in HK2s and hRPTECs. To verify the effect of *Leptospira* on E-cadherin expression, the E-cadherin protein levels in the HK2s and hRPTECs infected with pathogenic *L. santarosai* serovar Shermani (*L. santarosai*) and non-pathogenic *L. biflexa* serovar Patoc (*L. biflexa*) at a multiplicity of infection (MOI) of 100 were examined using western blotting. Compared with those in the control HK2s and hRPTECs (cultured in EMJH medium), the E-cadherin protein levels were significantly downregulated in the *L. santarosai*-infected HK2s and hRPTECs but non-significantly downregulated in the *L. biflexa*-infected HK2s and hRPTECs (Figure 3E,F). These findings indicate that rLRR20 and *L. santarosai* downregulated the E-cadherin levels in kidney cells.

### 2.3. rLRR20 Promotes the Activation and Nuclear Translocation of β-Catenin

**Figure 4:**
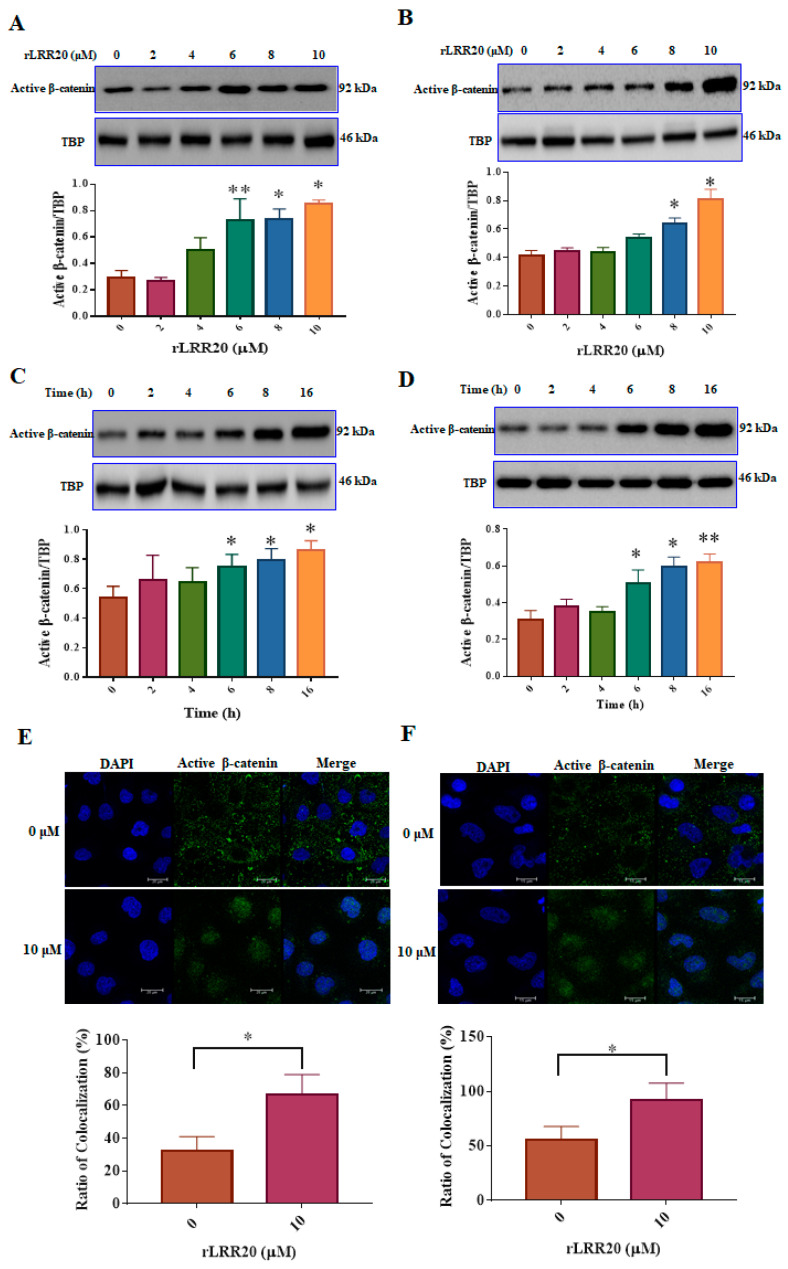
rLRR20 promotes the activation of β-catenin and its nuclear translocation. The nuclear fraction was prepared using the cytoplasmic and nuclear protein extraction kit (BRARZ106). Anti-activated β-catenin antibody was used to detect the expression level of activated β-catenin. TATA-box binding protein (TBP) was used as an internal control. Effect of treatment with different concentrations (0, 2, 4, 6, 8, and 10 μM) of rLRR20 for 8 h on the levels of activated β-catenin in the nucleus in HK2s (**A**) and hRPTECs (**B**). Effect of treatment with 10 μM rLRR20 for 0, 2, 4, 6, 8, or 16 h on the levels of activated β-catenin in the nucleus in HK2s (**C**) and hRPTECs (**D**). Effect of treatment with 10 μM rLRR20 and phosphate-buffered saline (control) for 8 h on the levels of activated β-catenin in HK2s (**E**) and hRPTECs (**F**) was examined using confocal microscopy. Mander’s overlap coefficients were calculated to determine the colocalization of activated β-catenin in the nucleus. Activated β-catenin (green); nucleus (blue). ** *p* < 0.01; * *p* < 0.05.

To examine the effect of rLRR20 on the downstream signaling of E-cadherin, the expression of β-catenin, a major E-cadherin intracellular domain-binding component, was examined using western blotting and confocal microscopy (Figure 4). The HK2s treated with various concentrations (0, 2, 4, 6, 8, and 10 μM) of rLRR20 for 8 h were lysed and subjected to immunoblotting. The cytoplasmic and nuclear fractions of the lysed cells were prepared using the cytoplasmic and nuclear protein extraction kit (#BRARZ106, BioTools). The regulatory effect of rLRR20 on β-catenin expression in the nuclear fraction was analyzed. The effects of rLRR20 on the levels of activated β-catenin, which is activated through dephosphorylation of Ser^37^ and Thr^41^, were examined [27]. Treatment with rLRR20 upregulated the activated β-catenin levels in HK2s (Figure 4A) and hRPTECs (Figure 4B). At high concentrations, rLRR20 significantly upregulated the nuclear levels of activated β-catenin (≥6 μM for HK2s and ≥8 μM for hRPTECs). Additionally, the activated β-catenin levels in the nuclear fraction of HK2s and hRPTECs treated with 10 μM rLRR20 for 0, 2, 4, 6, 8, or 16 h were examined using immunoblotting. Treatment with rLRR20 for 6 h significantly upregulated the activated β-catenin levels in HK2s (Figure 4C) and hRPTECs (Figure 4D). TATA-box binding protein (TBP) was used as an internal control in western blotting analysis. The nuclear translocation of activated β-catenin was examined using confocal microscopy (Figure 4E,F). For confocal microscopy analysis, the HK2s and hRPTECs were treated with 10 μM rLRR20 or PBS for 8 h. Mander’s overlap coefficients were calculated to determine the proportion of activated β-catenin colocalized in the nucleus (Figure 4E,F). Treatment with rLRR20 significantly upregulated the nuclear translocation of activated β-catenin (Figure 4).

### 2.4. rLRR20 Induces MMP7 Expression

Previous studies have indicated that MMP7 is a downstream target of β-catenin [5,7,11]. Microarray analysis revealed that treatment with rLRR20 upregulated the expression of *MMP7* (Figure 1D). As MMP7 is reported to degrade E-cadherin, the effect of rLRR20 on the crosstalk between E-cadherin, β-catenin, and MMP7 was examined [7]. The mRNA and protein levels of MMP7 in HK2s and hRPTECs incubated with various concentrations (0, 2, 4, 6, 8, and 10 μM) of rLRR20 for 8 h were measured. The cells and culture supernatants were used for measuring the mRNA and protein levels of MMP7, respectively. Compared with those in the control HK2s and hRPTECs, the *MMP7* mRNA levels were significantly upregulated in the rLRR20-treated HK2s (at rLRR20 concentrations ≥ 4 μM; Figure S3A) and hRPTECs (at rLRR20 concentrations ≥ 6 μM; Figure S3B), respectively. Additionally, the HK2s and hRPTECs were incubated with 10 μM rLRR20 for 0, 2, 4, 6, 8, or 16 h. The *MMP7* mRNA levels in the HK2s and hRPTECs treated with rLRR20 for ≥2 h were significantly higher than those in the control HK2s and hRPTECs (Figure S3C,D), respectively. The protein levels of active MMP7 in the culture supernatant of HK2s and hRPTECs treated with 0, 2, 4, 6, 8, and 10 μM rLRR20 for 8 h were measured. Compared with those in the control HK2s and hRPTECs, the active MMP7 protein levels were significantly upregulated at rLRR20 concentration ≥ 6 μM in the rLRR20-treated HK2s and hRPTECs (Figure S3E,F), respectively. Additionally, the active MMP7 protein levels in the HK2s and hRPTECs treated with rLRR20 for ≥4 h were significantly higher than those in the control HK2s and hRPTECs (Figure S3G,H), respectively. These findings indicate that rLRR20 dose-dependently and time-dependently upregulated the expression of active MMP7 (Figure S3).

**Figure 5:**
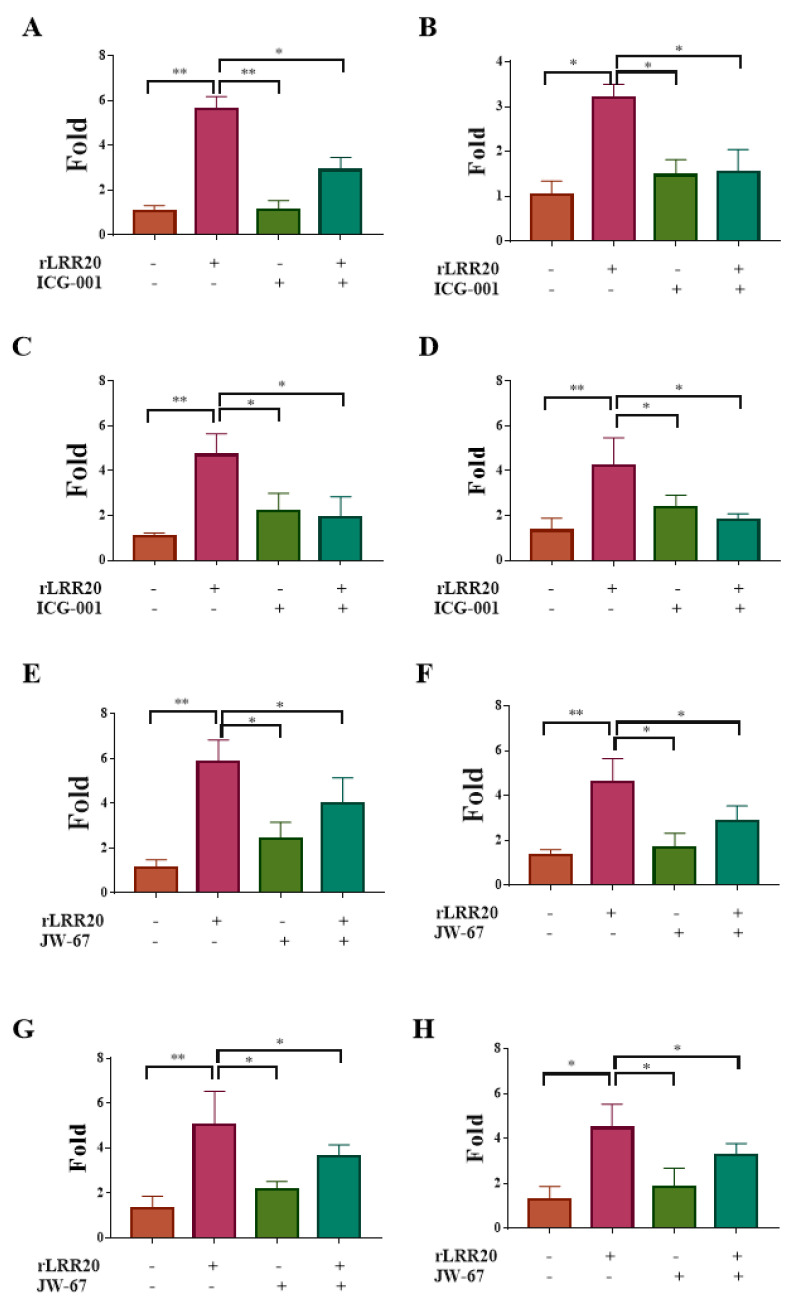
β-catenin inhibitors suppress rLRR20-induced MMP7 expression. Effect of the β-catenin inhibitors ICG-001 and JW-67 on rLRR20-induced MMP7 expression in HK2s and hRPTECs was examined. Effect of ICG-001 on the rLRR20-induced *MMP-7* mRNA levels in HK2s (**A**) and hRPTECs (**B**). Effect of ICG-001 on the rLRR20-induced MMP7 protein levels in HK2s (**C**) and hRPTECs (**D**). Effect of JW-67 on the rLRR20-induced *MMP7* mRNA levels in HK2s (**E**) and hRPTECs (**F**). Effect of JW-67 on the rLRR20-induced MMP7 protein levels in HK2s (**G**) and hRPTECs (**H**). ** *p* < 0.01; * *p* < 0.05.

To examine the correlation between E-cadherin, active MMP7, and β-catenin, the effect of the β-catenin-specific inhibitor ICG-001 (Table 2) on the expression of active MMP7 was examined. The HK2s and hRPTECs were incubated with rLRR20 (10 μM), ICG-001 (1 μM), or the combination of rLRR20/ICG-001 for 8 h. The mRNA and protein levels of *MMP7* in the cells and culture supernatant, respectively, were measured. Compared with those in the dimethyl sulfoxide (DMSO)-treated HK2s and hRPTECs, the *MMP7* mRNA levels were significantly upregulated in the rLRR20-treated HK2s and hRPTECs, respectively (Figure 5A,B). The *MMP7* mRNA levels in the ICG-001-treated HK2s and hRPTECs were similar to those in the DMSO-treated HK2s and hRPTECs, respectively (Figure 5A,B). Compared with those in the rLRR20-treated HK2s and hRPTECs, the *MMP7* mRNA levels were significantly downregulated in the rLRR20/ICG-001-treated HK2s and hRPTECs, respectively (Figure 5A,B). The active MMP7 protein levels in the rLRR20-treated HK2s and hRPTECs were significantly upregulated compared with those in the control HK2s and hRPTECs, respectively (Figure 5C,D). Compared with those in the DMSO-treated HK2s and hRPTECs, the active MMP7 protein levels were similar in the ICG-001-treated HK2s and hRPTECs (Figure 5C,D). The active MMP7 protein levels in the rLRR20/ICG-001-treated HK2s and hRPTECs were significantly downregulated compared with those in the rLRR20-treated HK2s and hRPTECs, respectively (Figure 5C,D). Similarly, another β-catenin-specific inhibitor JW-67 (Table 2) were used to further verify the correlation between E-cadherin, active MMP7, and β-catenin, and the results are similar to rLRR20/ICG-001-treated HK2s and hRPTECs (Figure 5E–H). These results indicated that ICG-001 and JW-67 inhibited the expression of active MMP7 in the rLRR20-treated HK2s and hRPTECs and that β-catenin was located upstream of MMP7 (Figure 5).

Next, the effect of *Leptospira* on the expression of active MMP7 was examined. The mRNA and protein levels of MMP7 in HK2s and hRPTECs incubated with pathogenic *L. santarosai* and non-pathogenic *L. biflexa* at an MOI of 100 were measured. Compared with those in the control HK2s and hRPTECs (cultured in EMJH medium), the *MMP7* mRNA levels were significantly upregulated in the rLRR20-treated and *L. santarosai*-treated HK2s and hRPTECs (Figure S4A,B), respectively. The *MMP7* mRNA levels in the *L. biflexa*-treated HK2s and hRPTECs were non-significantly upregulated compared with those in the control HK2s and hRPTECs (Figure S4A,B), respectively. The active MMP7 protein levels in the rLRR20-treated and *L. santarosai*-treated HK2s and hRPTECs were significantly upregulated when compared with those in the control HK2s and hRPTECs (Figure S4C,D). These findings indicated that *Leptospira* promoted active MMP7 protein expression in kidney cells (Figure S4).

### 2.5. rLRR20 Promotes the Degradation of E-Cadherin

**Figure 6:**
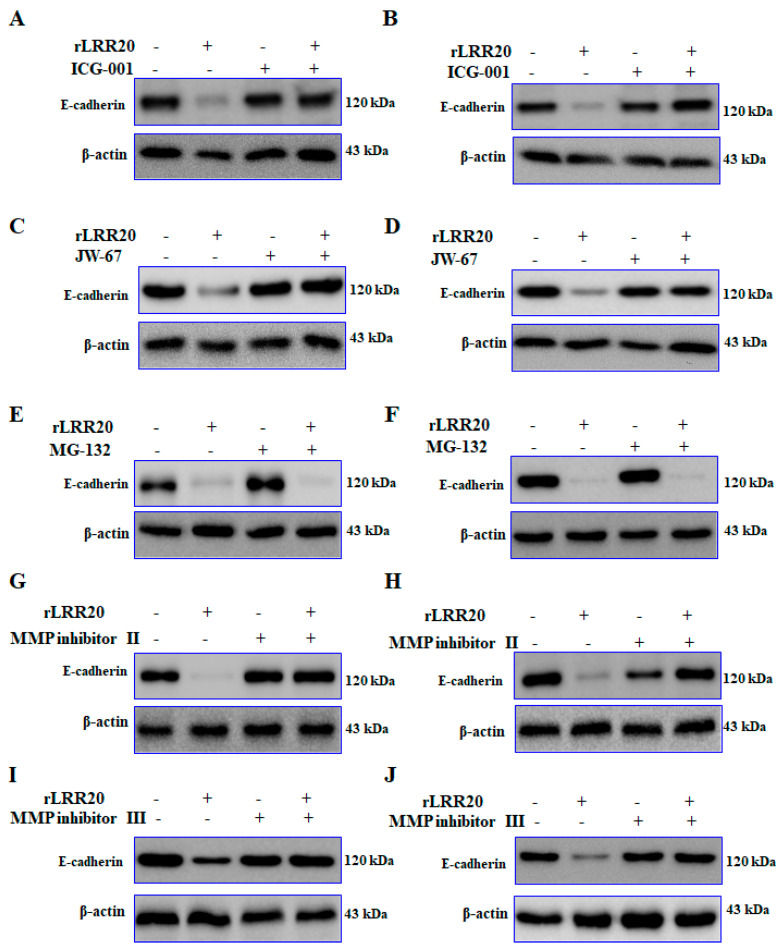
rLRR20 promotes the degradation of E-cadherin. rLRR20-induced degradation of E-cadherin was determined using various inhibitors. Effect of ICG-001, a β-catenin inhibitor, on the rLRR20-induced degradation of E-cadherin in HK2s (**A**) and hRPTECs (**B**). Effect of JW-67, a β-catenin inhibitor, on the rLRR20-induced degradation of E-cadherin in HK2s (**C**) and hRPTECs (**D**). Effect of MG-132, a proteasome inhibitor, on the rLRR20-induced degradation of E-cadherin in HK2s (**E**) and hRPTECs (**F**). Effect of MMP inhibitor II, an MMP7 inhibitor, on the rLRR20-induced degradation of E-cadherin in HK2s (**G**) and hRPTECs (**H**). Effect of MMP inhibitor III, an MMP-7 inhibitor, on the rLRR20-induced degradation of E-cadherin in HK2s (**I**) and hRPTECs (**J**). β-actin was used as the internal control.

The role of active MMP7 in the degradation of E-cadherin on the cell surface was examined. The E-cadherin protein in the HK2s and hRPTECs treated with various inhibitors were examined using western blotting. Compared with those in the control HK2s and hRPTECs, the E-cadherin protein was significantly downregulated in the rLRR20-treated HK2s and hRPTECs, respectively (Figure 6A,B). The E-cadherin protein in the ICG-001/rLRR20-treated HK2s and hRPTECs were similar in those in the control HK2 and hRPTECs, respectively (Figure 6A,B). The inhibitory effects of ICG-001 on the expression of E-cadherin in HK2s and hRPTECs were verified using JW-67. The E-cadherin protein in the rLRR20-treated HK2s and hRPTECs were significantly downregulated compared with those in the control HK2 and hRPTECs, respectively (Figure 6C,D). Compared with those in the control HK2s and hRPTECs, the E-cadherin proteins were similar in the JW-67/rLRR20-treated HK2s and hRPTECs, respectively (Figure 6C,D). These findings indicated that MMP7 expression promoted the degradation of E-cadherin and that ICG-001 and JW-67 inhibited the MMP7-mediated E-cadherin degradation. To examine the involvement of the proteasome in the degradation of E-cadherin on the cell surface, the cells were treated with the proteasome inhibitor MG-132. Co-treatment with rLRR20 and MG-132 downregulated the expression of E-cadherin in HK2s and hRPTECs (Figure 6E,F). These findings demonstrated that the degradation of E-cadherin was not dependent on the proteasome (Figure 6E,F). To confirm whether MMP7 directly degraded E-cadherin, the cells were treated with MMP7 inhibitors (MMP inhibitors II and III; Table 2). The E-cadherin protein in the rLRR20-treated HK2s and hRPTECs were significantly downregulated compared with those in the control HK2s and hRPTECs, respectively (Figure 6G,H). Compared with those in the control HK2s and hRPTECs, the E-cadherin proteins were similar in the rLRR20/MMP inhibitor II-treated HK2s and hRPTECs, respectively (Figure 6G,H). The E-cadherin protein in the rLRR20/MMP inhibitor III-treated HK2s and hRPTECs were similar to those in the control HK2s and hRPTECs, respectively (Figure 6I,J). These results further confirmed that MMP7 mediated the degradation of E-cadherin in the rLRR20-treated cells [35].

### 2.6. Activation of NF-κB by Degradation of Cell Surface E-Cadherin

**Figure 7:**
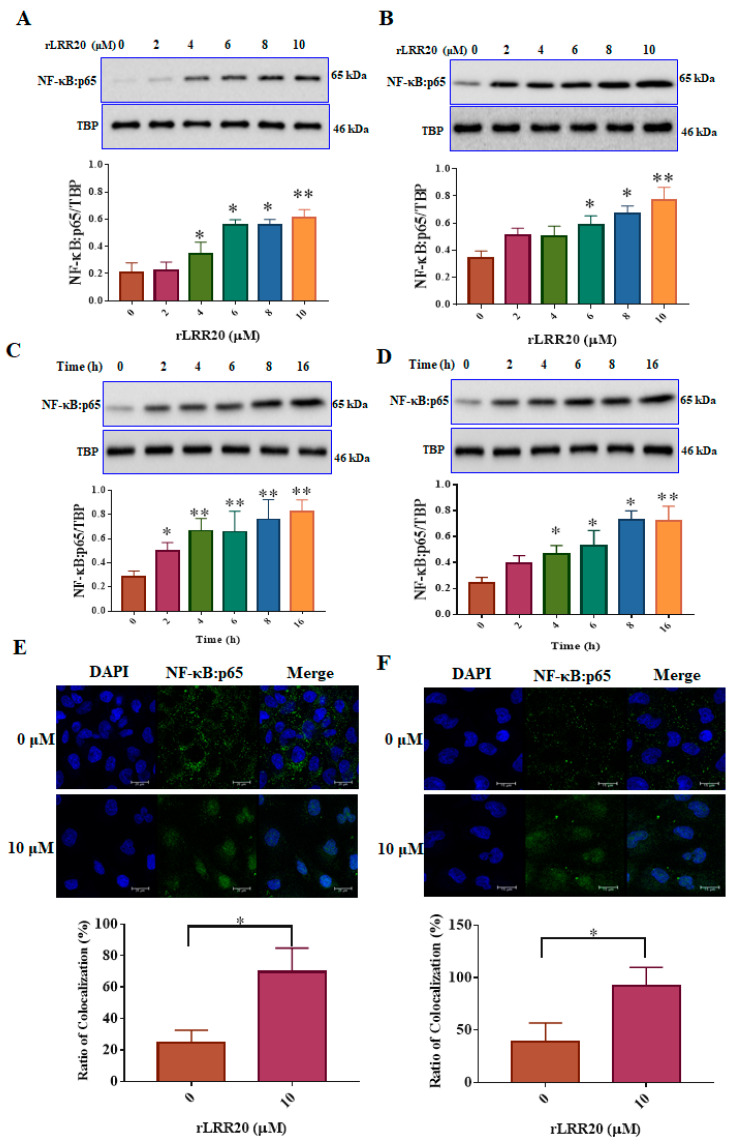
rLRR20 promotes the nuclear translocation of NF-κB:p65. The nuclear fraction was prepared using a cytoplasmic and nuclear protein extraction kit (BRARZ106). NF-κB:p65 was detected using the anti-p65 antibody. TATA-box binding protein (TBP) was used as the internal control. Effect of treatment with different concentrations of rLRR20 for 8 h on the levels of NF-κB:p65 in the nucleus in HK2s (**A**) and hRPTECs (**B**). Effect of treatment with 10 μM of rLRR20 for different durations on the NF-κB:p65 levels in the nucleus in HK2s (**C**) and hRPTECs (**D**). (**E**) Effect of treatment with 10 μM rLRR20 protein and phosphate-buffered saline for 8 h on the NF-κB:p65 levels in HK2s (**E**) and hRPTECs (**F**) was examined using confocal microscopy. Mander’s overlap coefficients were calculated to determine the colocalization of NF-κB:p65 with the nucleus. NF-κB:p65 (green); nucleus (blue). ** *p* < 0.01; * *p* < 0.05.

To further investigate the downstream signaling of MMP7-mediated E-cadherin degradation, the expression of NF-κB:p65, a major signal transduction component, was examined using western blotting and confocal microscopy (Figure 7). The nuclear fraction was isolated from the lysed rLRR20-treated HK2s and hRPTECs. The HK2s and hRPTECs were treated with various concentrations (0, 2, 4, 6, 8, and 10 μM) of rLRR20 for 8 h, lysed, and subjected to immunoblotting. The nuclear fraction was probed with an anti-p65 antibody to detect the expression of NF-κB:p65. Treatment with ≥4 and ≥6 μM rLRR20 significantly upregulated the expression of NF-κB:p65 in HK2s and hRPTECs, respectively (Figure 7A,B). The effects of treatment with 10 μM rLRR20 for 0, 2, 4, 6, 8, and 16 h on the protein levels of NF-κB:p65 in the HK2s and hRPTECs were examined. Treatment with rLRR20 for ≥2 and ≥4 h significantly upregulated the NF-κB:p65 protein levels in HK2s and hRPTECs, respectively (Figure 7C,D). TBP was used as an internal control in western blot analysis. Confocal microscopy analysis was performed to investigate the nuclear translocation of NF-κB:p65. The HK2s and hRPTECs were treated with 10 μM rLRR20 or PBS for 8 h, and results indicated that the nuclear translocation of NF-κB:p65 in HK2s and hRPTECs (Figure 7E,F). Mander’s overlap coefficients were calculated to determine the proportion of NF-κB:p65 colocalizing with the nucleus. Results indicated that rLRR20 significantly promoted the nuclear translocation of NF-κB:p65 (Figure 7).

### 2.7. rLRR20 Induces NGAL Expression

NGAL was reported to be an early biomarker and outcome predictor of leptospirosis-associated AKI [9]. In this study, microarray analysis revealed that rLRR20 upregulates the expression of *NGAL* (Figure 1E). To further investigate the regulatory effects of rLRR20 on the expression of NGAL, the HK2s and hRPTECs were treated with various concentrations (0, 2, 4, 6, 8, and 10 μM) of rLRR20 for different durations. Compared with those in the control HK2s and hRPTECs, the *NGAL* mRNA levels were significantly upregulated in the rLRR20-treated HK2s (at rLRR20 treatment concentrations of ≥6 μM) and hRPTECs (at rLRR20 treatment concentrations of ≥8 μM), respectively (Figure S5A,B). Additionally, the HK2s and hRPTECs were incubated with 10 μM rLRR20 for different durations. Treatment with 10 μM rLRR20 for ≥6 h significantly upregulated the *NGAL* mRNA levels in the HK2s and hRPTECs (Figure S5C,D). The NGAL protein levels in the HK2s and hRPTECs treated with ≥8 μM rLRR20 were higher than those in the control HK2s and hRPTECs, respectively (Figure S5E,F). Additionally, the HK2s and hRPTECs were incubated with 10 μM rLRR20 for 0, 2, 4, 6, 8, 10, and 16 h. Compared with those in the control HK2s and hRPTECs, the NGAL protein levels were significantly upregulated in the HK2s and hRPTECs treated with rLRR20 for ≥6 h, respectively (Figure S5G,H). These findings indicated that rLRR20 dose-dependently and time-dependently upregulated the expression of NGAL.

**Figure 8:**
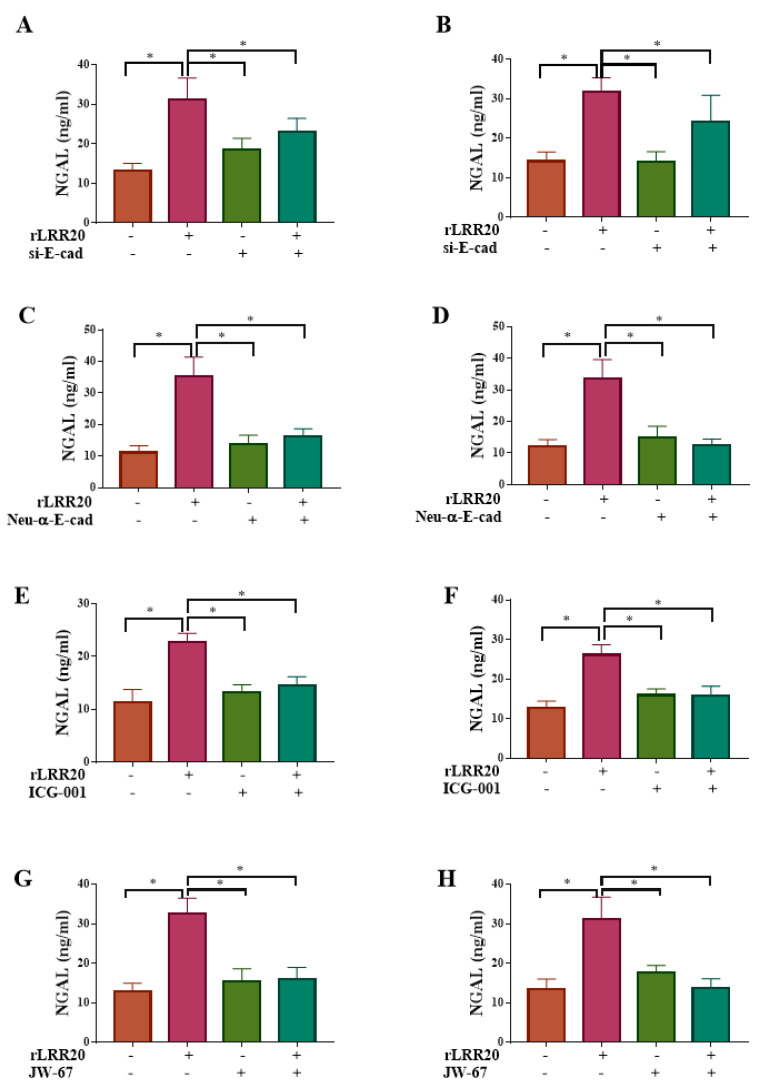
rLRR20 regulates NGAL expression through the E-cadherin/β-catenin pathway. rLRR20-mediated regulation of NGAL expression through the E-cadherin/β-catenin pathway was determined using various inhibitors. Effect of short interfering RNA against E-cadherin (si-E-cad) on NGAL expression in HK2s (**A**) and hRPTECs (**B**). Effect of Neu-α-E-cad antibody on NGAL expression in HK2s (**C**) and hRPTECs (**D**). Effect of ICG-001, a β-catenin inhibitor, on NGAL expression in HK2s (**E**) and hRPTECs (**F**). JW-67, a β-catenin inhibitor, on NGAL expression in HK2s (**G**) and hRPTECs (**H**). Dimethyl sulfoxide (DMSO) was used as the control. * *p* < 0.05.

The regulatory effect of rLRR20 on NGAL protein expression was further confirmed by silencing the expression of *E-cadherin* using si-E-cad. The NGAL protein levels in the rLRR20-treated HK2s and hRPTECs were significantly upregulated when compared with those in the DMSO-treated HK2s (Figure 8A) and hRPTECs (Figure 8B), respectively. Compared with those in the rLRR20-treated HK2s and hRPTECs, the NGAL protein levels were significantly downregulated in the si-E-cad/rLRR20 HK2s (Figure 8A) and hRPTECs (Figure 8B), respectively. These findings indicate that rLRR20-induced NGAL protein expression was dependent on E-cadherin. To further investigate the role of E-cadherin in rLRR20-induced NGAL expression, the cells were treated with the Neu-α-E-cad. The NGAL protein levels in the rLRR20-treated HK2s and hRPTECs were significantly upregulated when compared with those in the DMSO-treated HK2s (Figure 8C) and hRPTECs (Figure 8D), respectively. Compared with those in the rLRR20-treated HK2s and hRPTECs, the NGAL protein levels were significantly downregulated in the Neu-α-E-cad/rLRR20-treated HK2s (Figure 8C) and hRPTECs (Figure 8D), respectively. These findings further confirmed that rLRR20-induced NGAL protein expression was dependent on E-cadherin. Furthermore, the correlation between β-catenin and NGAL was examined using the β-catenin inhibitor ICG-001. The NGAL protein levels in the ICG-001/rLRR20-treated HK2s and hRPTECs were similar to those in the control HK2s and hRPTECs, respectively, but significantly lower than those in the rLRR20-treated HK2s (Figure 8E) and hRPTECs (Figure 8F), respectively. Thus, ICG-001 significantly inhibited rLRR20-induced NGAL protein expression (Figure 8E,F). To verify the inhibitory effects of ICG-001 on NGAL expression, the cells were treated with JW-67. The NGAL protein levels in the JW-67/rLRR20-treated HK2s and hRPTECs were similar to those in the control HK2s and hRPTECs, respectively, but significantly lower than those in the rLRR20-treated HK2s (Figure 8G) and hRPTECs (Figure 8H), respectively.

**Figure 9:**
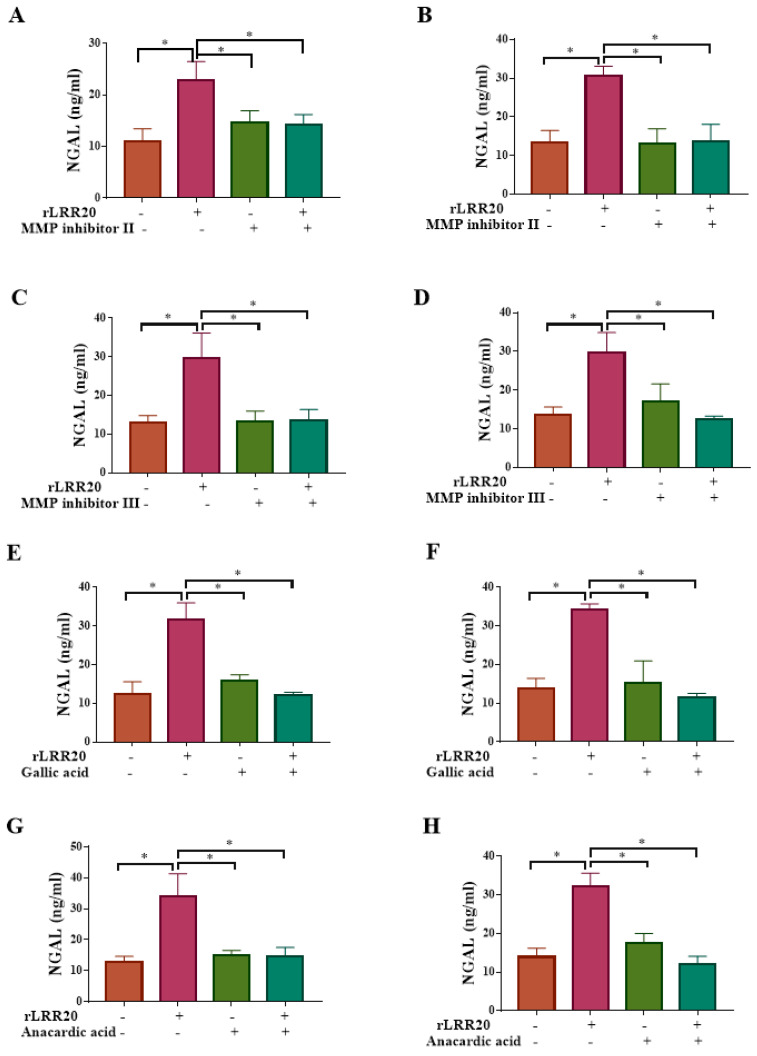
rLRR20 promotes the expression of NGAL by activating the NF-κB pathway. rLRR20-induced upregulation of NGAL through the NF-κB signaling pathway was demonstrated using various inhibitors. Effect of MMP inhibitor II, an MMP7 inhibitor, on the NGAL levels in HK2s (**A**) and hRPTECs (**B**). Effect of MMP inhibitor III, an MMP7 inhibitor, on the NGAL levels in HK2s (**C**) and hRPTECs (**D**). Effect of gallic acid on the NGAL levels in HK2s (**E**) and hRPTECs (**F**). Effect of anacardic acid, a NF-κB:p65 inhibitor, on the NGAL levels in HK2s (**G**) and hRPTECs (**H**). Dimethyl sulfoxide (DMSO) was used as the control. * *p* < 0.05.

Moreover, the HK2s and hRPTECs were treated with MMP7 inhibitors to examine the correlation between MMP7 and NGAL (Table 2). The NGAL protein levels in the MMP inhibitor II/rLRR20-treated HK2s and hRPTECs were similar to those in the control HK2s and hRPTECs, respectively, but significantly lower than those in the rLRR20-treated HK2s (Figure 9A) and hRPTECs (Figure 9B), respectively. Similarly, the NGAL protein levels in the MMP inhibitor III/rLRR20-treated HK2s and hRPTECs were similar to those in the control HK2s and hRPTECs, respectively, but significantly lower than those in the rLRR20-treated HK2s (Figure 9C) and hRPTECs (Figure 9D), respectively. These findings indicate that the MMP7 inhibitors significantly inhibited rLRR20-induced NGAL expression (Figure 9A–D). Furthermore, the correlation between NF-κB:p65 and NGAL protein expression in HK2s and hRPTECs were examined using the NF-κB:p65 inhibitors gallic acid and anacardic acid (Table 2). The NGAL protein levels in the gallic acid/rLRR20-treated HK2s and hRPTECs were similar to those in the DMSO-treated HK2s and hRPTECs, respectively, but significantly lower than those in the rLRR20-treated HK2s (Figure 9E) and hRPTECs (Figure 9F), respectively. Additionally, the NGAL protein levels in the anacardic acid/rLRR20-treated HK2s and hRPTECs were similar to those in the DMSO-treated HK2s and hRPTECs, respectively, but significantly lower than those in the rLRR20-treated HK2s (Figure 9G) and hRPTECs (Figure 9H), respectively. These findings indicated that rLRR20 promotes the expression of NGAL through the E-cadherin/β-catenin and NF-κB signaling pathways.

Next, the effect of *Leptospira* on the expression of NGAL was examined. The NGAL protein levels in the HK2s and hRPTECs treated with pathogenic *L. santarosai* and non-pathogenic *L. biflexa* were examined. The *NGAL* mRNA levels in the rLRR20/*L. santarosai*-treated HK2s and hRPTECs were significantly upregulated compared with those in the control HK2s (Figure S6A) and hRPTECs (Figure S6B), respectively. Similarly, the *NGAL* mRNA levels in the *L. biflexa*-treated hRPTECs were significantly upregulated when compared with those in the control hRPTECs (Figure S6B). Compared with those in the control HK2s and hRPTECs, the NGAL levels were significantly upregulated in the rLRR20/*L. santarosai*-treated HK2 (Figure S6C) and hRPTECs (Figure S6D), respectively. These findings indicate that *Leptospira* promoted NGAL expression in kidney cells (Figure S6).

## 3. Discussion

The first step of *Leptospira* infection involves the attachment of the pathogen to the host cells [36]. In this study, treatment with rLRR20, which colocalized with E-cadherin on the cell surface, dose-dependently and time-dependently downregulated the expression of E-cadherin (Figure 2). A previous study also confirmed that the interaction of *Leptospira* and cadherin molecules in the host cells and the LIC10831 from pathogenic *Leptospira* interacted with E- and VE-cadherin [16,17]. However, treatment with rLRR20 did not affect the *E-cadherin* mRNA levels, which indicated that rLRR20 regulates E-cadherin at the protein level (Figure S2 and Figure 2). Therefore, the interaction between E-cadherin and rLRR20 was blocked using the Neu-α-E-cad to examine the effect of Neu-α-E-cad on rLRR20-induced E-cadherin degradation. The E-cadherin protein levels in the Neu-α-E-cad-treated HK2s and hRPTECs were higher than those in the rLRR20-treated HK2s and hRPTECs (Figure 3A,B). Additionally, the expression of *E-cadherin* was knocked down using si-E-cad. Compared with those in the rLRR20-treated HK2s and hRPTECs, the E-cadherin levels were significantly downregulated in the rLRR20/si-E-cad HK2s and hRPTECs, respectively (Figure 3C,D). This indicated that E-cadherin is the binding target of rLRR20 and that the regulation of E-cadherin plays a vital role in leptospirosis (Figure 3E,F). To explore the regulatory effects of rLRR20 on E-cadherin expression, the levels of β-catenin, which is the downstream signaling component of E-cadherin, were examined. Treatment with rLRR20 promoted β-catenin activation and the nuclear translocation of activated β-catenin (Figure 4). Previous studies have reported that *MMP7* is a target gene of β-catenin [5,7,11]. MMP7 is reported to be a novel biomarker of kidney injury and fibrosis [5,7]. rLRR20 stimulated the expression of active MMP7 (Figure S3 and Figure 5). Previous studies have also demonstrated that active MMP7 degrades surface E-cadherin in kidney epithelial cells under kidney injury conditions [7,12]. In this study, treatment with rLRR20 markedly downregulated the expression of E-cadherin in HK2s and hRPTECs, indicating that active MMP7 may promote the degradation of E-cadherin (Figure S3 and Figure 5). To verify this, the cells were treated with the following inhibitors: ICG-001, JW-67, MG-132, MMP inhibitor II, and MMP inhibitor III (Figure 6). The proteasome inhibitor MG-132 did not suppress the degradation of E-cadherin, indicating that MMP7 plays a major role in the degradation of E-cadherin (Figure 7E,F). These results demonstrate that rLRR20 induces the downstream signaling component MMP7 to degrade surface E-cadherin. *Leptospira* upregulated the expression of MMP7 in kidney cells (Figure S4). Interestingly, treatment with a high concentration of rLRR20 (>4 μM) and prolonged incubation period (>6 h) induced feedback degradation of surface E-cadherin (Figure 2B,C), which may prevent *Leptospira* colonization. Interestingly, previous studies also demonstrated that MMP7 degrades E-cadherin [35,37]. *Leptospira* infection upregulated the MMP7 expression to degrade E-cadherin, and the degradation of E-cadherin could help host cells against the infection of *Leptospira*, and the releasing of the EC1 domain could further help host cells against the infection of *Leptospira* through interaction with an LRR20 virulence factor.

**Figure 10:**
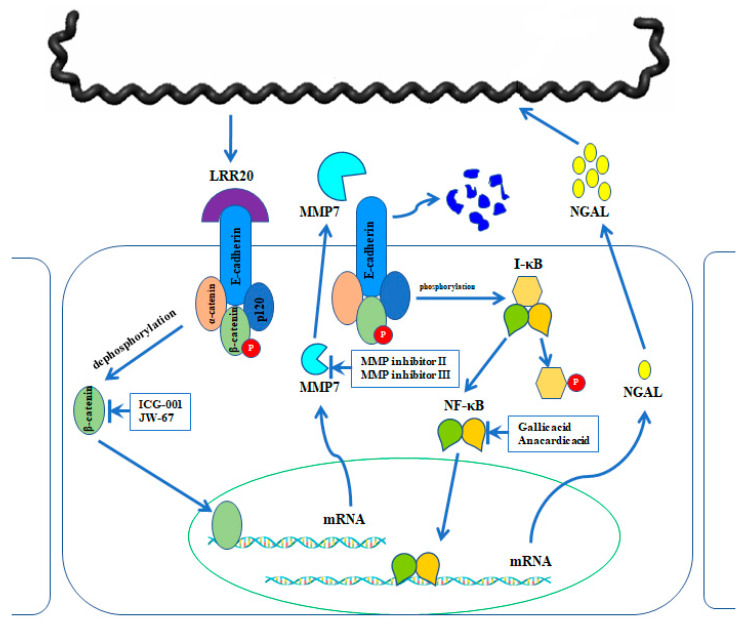
Proposed working model for the signal transduction crosstalk between E-cadherin/β-catenin and NF-κB signaling pathways. *Leptospira* colonizes the kidney epithelial cells through various adhesins. Recombinant LRR20 (rLRR20) protein interacts with E-cadherin and consequently activates β-catenin. The nuclear translocation of activated β-catenin promotes the expression of its target genes, including *MMP7*. Subsequently, MMP7 is secreted to the extracellular region. The expression and secretion of MMP7 promote the degradation of E-cadherin on the cell surface and downregulate the cell surface levels of E-cadherin. Meanwhile, the degradation of E-cadherin on the cell surface induces the activation of the NF-κB:p65 signal transduction pathway, which subsequently promotes the expression of downstream target gene *NGAL*. The inhibitors used to inhibit the specific targets are shown.

To prevent *Leptospira* colonization, kidney epithelial cells have developed other mechanisms. NGAL exerts antibacterial effects by sequestering iron-containing siderophores [25]. Additionally, NGAL is a sensitive biomarker for AKI. The expression of NGAL is upregulated in human epithelial cells under bacterial infection and inflammatory conditions [25]. In this study, rLRR20 upregulated the expression of NGAL (Figure 9 and Figure 10). To further clarify the mechanism underlying rLRR20-induced NGAL expression, the effect of rLRR20 on the levels of the NGAL transcription factor NF-κB:p65 was examined (Figure 7) [38]. Treatment with rLRR20 significantly upregulated the levels of NF-κB:p65. Additionally, rLRR20 promoted the nuclear translocation of NF-κB:p65 at high treatment doses (Figure 5C). These findings demonstrated that rLRR20 upregulates NGAL expression by stimulating the expression of NF-κB:p65 (Figure 7). However, the correlation between MMP7-mediated E-cadherin degradation and NGAL upregulation has not been elucidated. A previous review paper reported that the regulation between β-catenin and the NF-κB and the regulation including positive and negative regulations in different cell types and different stimulation agents [39]. The findings of this study indicated that MMP7 might play crucial roles in regulating E-cadherin, β-catenin, and NF-κB:p65 transcriptional activity (Figure 8 and Figure 9). In leptospirosis, rLRR20 stimulated the expression of MMP7 and NGAL only at concentrations higher than 6 μM. Therefore, infection from increased numbers of *Leptospira* (severe *Leptospira* invasion) could induce kidney injury through the outer membrane virulence factor LRR20.

rLRR20 interacts with E-cadherin and activates downstream β-catenin, which results in the nuclear translocation of activated β-catenin and the consequent activation of MMP7 expression. MMP7 is secreted and further degrades E-cadherin on the cell surface. MMP7-mediated downregulation of surface E-cadherin activates NF-κB:p65, which results in the nuclear translocation of NF-κB:p65 and the subsequent activation of NGAL expression. NGAL, which is upregulated in human kidney epithelial cells during bacterial infection and inflammatory conditions, is a biomarker for AKI. This study proposed a novel model (Figure 10) for the crosstalk between the two signal transduction pathways and the mechanism involved in the protection of the host cells against bacterial colonization and infection. The upregulated expression of MMP7 and NGAL may be the mechanism underlying leptospirosis-induced kidney injury, which involves the membrane components of *Leptospira*.

## 4. Materials and Methods

### 4.1. Protein Purification

The rLRR20 (LSS_11580) protein was purified following the protocols of a previous study with minor modifications [40]. Briefly, the pRSET-LRR20 construct was transformed into the expression host ClearColi BL21(DE3) (Lucigen, Middleton, WI, USA). The recombinant bacterial cells were cultured in Luria broth medium containing 50 μg/mL ampicillin. To induce the expression of the proteins, the cells were incubated with 1 mM isopropyl β-D-1-thiogalactopyranoside at 16 °C for 16 h. rLRR20 was purified using a Histrap column (GE Healthcare Life Sciences, Chalfont, PA, USA) with 0–300 mM imidazole gradient. The fractions were subjected to sodium dodecyl sulfate-polyacrylamide gel electrophoresis (SDS-PAGE) using a 15% gel. The presence of rLRR20 was confirmed using western blotting with the anti-His-tag antibodies (66005-1-Ig; Proteintech, Rosemont, IL, USA). The purified rLRR20 was dialyzed against phosphate-buffered saline (PBS) to remove imidazole and stored at −80 °C before application.

### 4.2. Cell Culture, RNA Extraction, and Quantitative Real-Time Polymerase Chain Reaction (qRT-PCR)

Human kidney 2 cells (HK2s; #CRL-2190) and human renal proximal tubular epithelial cells (hRPTECs)/TERT1 (#CRL-4031) were purchased from the American Type Culture Collection (ATCC, VA, USA). The cells were cultured in a medium described in a previous study [40,41]. In brief, HK2s were cultured in DMEM/Ham’s F12 medium supplemented with 5% (*w*/*v*) FCS, 2 mM glutamine, 20 mM HEPES (pH 7.0), 0.4 μg/mL hydrocortisone, 5 μg/mL insulin, 5 μg/mL transferrin, and 28.9 μM sodium selenite. hRPTECs were cultured in DMEM/Ham’s F12 medium supplemented with 5% (*w*/*v*) FCS, 4 mM L-glutamine, 10 mM HEPES buffer, 5 pM triiodothyronine, 10 ng/mL recombinant human EGF, 3.5 μg/mL ascorbic acid, 5 μg/mL transferrin, 5 μg/mL insulin, 25 ng/mL prostaglandin E1, 25 ng/mL hydrocortisone, 8.65 ng/mL sodium selenite, and 100 μg/mL G418. The cells were cultured at 37 °C in a humidified atmosphere of 5% (*v*/*v*) CO_2_. Serum-starved HK2s and hRPTECs (2 × 10^7^) for 16 h were incubated with various concentrations of rLRR20 for different durations. Additionally, the rLRR20-treated HK2s and hRPTECs were cultured in the absence or presence of the following inhibitors: ICG-001 (Merck, Kenilworth, NJ, USA) [28], JW-67 (Merck), MMP inhibitor II (Merck) [5], MMP inhibitor III (Merck), MG-132 (Merck) [34], gallic acid (Merck), anacardic acid (Merck). Total RNA was extracted from the HK2s and hRPTECs using TRIzol reagent (Invitrogen, Carlsbad, CA, USA), following the manufacturer’s instructions [10]. qRT-PCR analysis was performed using an *ABI ViiA7* qRT-PCR system (Applied Biosystems, Waltham, MA, USA). The TaqMan primers used in the qRT-PCR analysis are listed in Table 1.

### 4.3. Bacterial Culture

*Leptospira biflexa* serovar Patoc (ATCC number 23582; non-pathogenic species) and *L. santarosai* serovar Shermani str. LT821 (ATCC number 43286; pathogenic species) were purchased from ATCC. The bacterial cells were cultured at 28 °C under aerobic conditions in the Ellinghausen–McCullough–Johnson–Harris (EMJH) medium, following the protocols described in a previous study [42]. The density of the bacterial culture was determined using a CASY-Model TT cell counter and analyzer (Roche Innovatis AG, Casy-Technology, Reutlingen, Germany). The pathogenic and non-pathogenic *Leptospira* were incubated with HK2s and hRPTECs at a multiplicity of infection (MOI) of 100 for 8 h. The protein levels of E-cadherin, active MMP7, and NGAL in the culture supernatant were analyzed.

### 4.4. Microarray Analysis

Total RNA was amplified, labeled, and hybridized to the Clariom D Assay chips (Thermo Fisher Scientific, Waltham, MA, USA), following the manufacturer’s instructions. Microarray data were submitted to the National Center for Biotechnology Information Gene Expression database (accession number: GSE155061). Differentially expressed genes with a fold change >2 and *p*-value < 0.05 were classified into functional groups.

### 4.5. Enzyme-Linked Immunosorbent Assay (ELISA)

The serum-starved HK2s and hRPTECs were treated with different concentrations of rLRR20 or bacterial for different durations. The supernatant was collected to analyze the levels of active MMP7 and NGAL using the human MMP7 (active) ELISA Kit (Cat # ARG82010, Arigo, Taiwan) and human NGAL quantikine ELISA kit (Cat # DLCN20, R&D Systems, Minneapolis, MN, USA), respectively, following the manufacturer’s instructions.

### 4.6. Confocal Microscopy

HK2s and hRPTECs were incubated with 10 μM of rLRR20, and the cells were washed, fixed, permeabilized, and incubated with the following primary antibodies: anti-E-cadherin (ab133597; Abcam Cambridge, MA, USA), anti-His-tag (66005-1-Ig; Proteintech, Rosemont, IL, USA), anti-active β-catenin (#8814; Cell Signaling, Danvers, MA, USA), and anti-p65 (ab16502; Abcam, Cambridge, MA, USA) antibodies. Next, the cells were incubated with Alexa488-conjugated and Alexa594-conjugated anti-rabbit or anti-mouse secondary antibodies (Research Diagnostics Inc., Mount Olive, NJ, USA) and subjected to confocal microscopy. The nuclei were stained using 4′,6-diamidino-2-phenylindole (DAPI), and the cells were imaged using a confocal laser scanning microscope (TCS-SP8-X, Leica, Wetzlar, Germany).

### 4.7. Western Blotting

The treated HK2s and hRPTECs were washed thrice with PBS and lysed directly using Mem-PER plus membrane protein extraction kit for membrane protein extraction (Cat# 89842, Thermo Fisher Scientific) supplemented with Halt protease and phosphatase inhibitors (Cat# 78446, Thermo Fisher Scientific). Cytoplasmic and nuclear fractions were prepared using a cytoplasmic and nuclear protein extraction kit (BRARZ106, BioTools Co. Ltd., Taiwan), following the manufacturer’s instructions. The concentration of proteins was determined using the Bradford method. The proteins were resolved using SDS-PAGE. The resolved proteins were transferred to a polyvinyl difluoride membrane. The membrane was incubated with the indicated primary antibodies, followed by incubation with horseradish peroxidase-conjugated secondary antibodies in Tris-buffered saline with 5% (*w*/*v*) milk or 1% (*w*/*v*) bovine serum albumin. The primary antibodies used in this study including anti-E-cadherin (ab238099; Abcam, Cambridge, MA, USA), anti-beta actin antibody (ab8227; Abcam, Cambridge, MA, USA), anti-active β-catenin (#8814; Cell Signal, Danvers, MA, USA), anti-p65 (ab16502; Abcam, Cambridge, MA, USA), and anti-TBP antibodies (ab28175; Abcam, Cambridge, MA, USA). Immunoreactive signals were visualized using an enhanced chemiluminescent detection reagent (GE Healthcare Life Sciences).

### 4.8. Statistical Analyses

At least three independent experiments were performed. The variables are expressed as mean ± standard error of the mean. The data were analyzed using Student’s *t*-test or one-way analysis of variance. The significant differences were considered at *p* < 0.05. All statistical analyses were performed using GraphPad Prism 5.1 (GraphPad, La Jolla, CA, USA).

## Figures and Tables

**Table 1:** Primers used in this study.

Primers		
Name	Forward Sequence (5′→3′)	Reverse Sequence (5′→3′)
LRR20WT	ctcgagatgacaaagtctcactctcctataaaac	ggtacctcaaaaatcaatattcgtattcgg
si-E-cad	L-003877-00-0005 (Dharmacon)	
E-cadherin (CDH1)	Hs01023895_m1 (Thermo Fisher Scientific)	
β-catenin (CTNNB1)	Hs00355045_m1 (Thermo Fisher Scientific)	
MMP7	Hs01042796_m1 (Thermo Fisher Scientific)	
NFκB (RELA)	Hs01042014_m1 (Thermo Fisher Scientific)	
GAPDH	Hs99999905_m1 (Thermo Fisher Scientific)	

**Table 2:** Inhibitors used in this study.

Target	Inhibitor	Function	Product Number	Supplier	References
β-catenin	ICG-001	Bind to CREB binding protein (CBP)	CAS 847591-62-2	Merck	[28]
	JW-67	Bind to β-catenin destruction complex (GSK-3β/AXIN/APC) to induce β-catenin degradation	CAS 442644-28-2	Merck	[29]
MMP7	MMP inhibitor II	Inhibit to matrix metalloproteinases	CAS 203915-59-7	Merck	[5,30]
	MMP inhibitor III	Inhibit to matrix metalloproteinases	CAS 927827-98-3	Merck	[31]
NF-κB:p65	Gallic acid	Prevents p65 acetylation	CAS 5995-86-8	Merck	[32]
	Anacardic acid	Inhibits p65 acetylation	CAS 16611-84-0	Merck	[33]
Proteasome	MG-132	Potent, reversible proteasome inhibitor disrupts RANKL signaling	CAS 1211877-36-9	Merck	[34]

## Data Availability

The data presented in this study are openly available in the NCBI Gene Expression Omnibus under reference numbers GSE155061.

## Supplementary Materials

The following are available online at https://www.mdpi.com/article/10.3390/ijms222313132/s1.
