# Supplementary material for: Crosstalk between E-Cadherin/β-Catenin and NF-κB Signaling Pathways: The Regulation of Host-Pathogen Interaction during Leptospirosis
Source: Int J Mol Sci. 2021 Dec 4;22(23):13132. doi: 10.3390/ijms222313132 (PMC8658460; doi:10.3390/ijms222313132)

# Supporting Information

## Crosstalk between E-cadherin/β-catenin and NF-κB Signaling Pathways: The Regulation of Host-Pathogen Interaction during Leptospirosis

Shen-Hsing Hsu[1,*], Li-Fang Chou[1], Chung-Hung Hong[1], Ming-Yang Chang[1], Chung-Ying Tsai[1], Ya-Chung Tian[1], Huang-Yu Yang[1], Chih-Wei Yang[1,*]

[1]Department of Nephrology, Kidney Research Center, Chang Gung Memorial Hospital, Chang Gung University College of Medicine, 5 Fu-Shing St. Taoyuan 33333, Taiwan

*For correspondence: Shen-Hsing Hsu (d938208@gmail.com) and Chih-Wei Yang (cwyang@ms1.hinet.net)

Short title: Roles of MMP7 and NGAL in Leptospirosis

## Supporting figure legends and table content

**Figure S1. Purification of rLRR20 and recognition of LRR20 in pathogenic and non-pathogenic *Leptospira* species.** (A) Blot image of the purified rLRR20 band resolved using a 15% gel. (B) Detection of LRR20 in pathogenic and non-pathogenic *Leptospira* species. Probing with the anti-rLRR20 antibody detected the presence of the virulence factor in pathogenic *Leptospira* but not in non-pathogenic *Leptospira*.

**Figure S2. rLRR20 downregulates E-cadherin expression on the cell surface.** Effect of treatment with different doses (0, 2, 4, 6, 8, and 10 μM) of rLRR20 for 8 h on the *E-cadherin* mRNA levels in

HK2s (A) and hRPTECs (B). Effect of treatment with 10 μM rLRR20 for 0, 2, 4, 6, 8, or 16 h on the *E-cadherin* mRNA levels in HK2s (C) and hRPTECs (D).

**Figure S3. rLRR20 stimulates MMP7 expression.** Concentration curves (0, 2, 4, 6, 8, and 10 μM) and time courses (0, 2, 4, 6, 8, and 16 h) of rLRR20-regulated MMP7 expression in HK2s and hRPTECs. Effect of treatment with different doses of rLRR20 for 8 h on the *MMP7* mRNA levels in HK2s (A) and hRPTECs (B). Effect of treatment with 10 μM rLRR20 for different durations on the *MMP7* mRNA levels in HK2s (C) and hRPTECs (D). Effect of treatment with different doses of rLRR20 for 8 h on the active MMP7 protein levels in HK2s (E) and hRPTECs (F). Effect of treatment with 10 μM rLRR20 for different durations on the active MMP7 protein levels in HK2s (G) and hRPTECs (H). $^{**}p < 0.01$; $^{*}p < 0.05$.

**Figure S4. *Leptospira* species promote active MMP7 expression.** Pathogenic and non-pathogenic *Leptospira* species were used to infect the HK2s and hRPTECs. Effect of Ellinghausen–McCullough–Johnson–Harris (EMJH) medium, recombinant LRR20 (rLRR20), *L. biflexa*, and *L. santarosai* on the *MMP7* mRNA levels in HK2s (A) and hRPTECs (B). Effect of EMJH medium, rLRR20, *L. biflexa*, and *L. santarosai* on the active MMP7 protein levels in HK2s (C) and hRPTECs (D). $^{*}p < 0.05$.

**Figure S5. rLRR20 promotes NGAL expression.** Concentration curves and time courses of rLRR20-mediated regulation of NGAL expression in HK2s andhRPTECs. Effect of treatment with different concentrations (0, 2, 4, 6, 8, and 10 μM) of rLRR20 for 8 h on the *NGAL* mRNA levels in

HK2s (A) and hRPTECs (B). Effect of treatment with 10 μM rLRR20 for 0, 2, 4, 6, 8, and 16 h on the *NGAL* mRNA levels in HK2s (C) and hRPTECs (D). Effect of treatment with different concentrations (0, 2, 4, 6, 8, and 10 μM) of rLRR20 for 8 h on the NGAL protein levels in HK2s (E) and hRPTECs (F). Effect of treatment with 10 μM rLRR20 for 0, 2, 4, 6, 8, and 16 h on the NGAL protein levels in HK2s (G) and hRPTECs (H). $**p < 0.01$; $*p < 0.05$.

**Figure S6. *Leptospira* species promote NGAL expression.** Pathogenic and non-pathogenic *Leptospira* species were used to infect the HK2s and hRPTECs. Effect of Ellinghausen–McCullough–Johnson–Harris (EMJH) medium, recombinant LRR20 (rLRR20), *L. biflexa*, and *L. santarosai* on the *NGAL* mRNA levels in HK2s (A) and hRPTECs (B). Effect of EMJH medium, rLRR20, *L. biflexa*, and *L. santarosai* on the NGAL protein levels in HK2s (C) and hRPTECs (D). $*p < 0.05$.

**Table S1. The gene IDs of the heatmap.** The regulation of the genes under the treatment of rLRR20 in HK2 cells were listed according the order of the heatmap and the fold change (log2 ratio) and the p value of these genes were listed.

# Table S1. The gene IDs of the heatmap.

| HeatMap Number | ID | Gene Symbol | LRR20 vs Control log2 ratio | p value | HeatMap Number | ID | Gene Symbol | LRR20 vs Control log2 ratio | p value | HeatMap Number | ID | Gene Symbol | LRR20 vs Control log2 ratio | p value | HeatMap Number | ID | Gene Symbol | LRR20 vs Control log2 ratio | p value |
|---|---|---|---|---|---|---|---|---|---|---|---|---|---|---|---|---|---|---|---|
| 1 | TC0100013722.hg.1 | SNORA63 | -1.03 | 0.005 | 72 | TC0100013465.hg.1 | | 1.03 | 0.018 | 143 | TC2000009294.hg.1 | skoyva | 1.09 | 0.000 | 214 | TC1900007311.hg.1 | dawkee | 1.21 | 0.016 |
| 2 | TC0300011714.hg.1 | | -1.24 | 0.002 | 73 | TC0800008322.hg.1 | gulu | 1.08 | 0.028 | 144 | TC0100008215.hg.1 | | 1.20 | 0.015 | 215 | TC0300006603.hg.1 | stardawbo | 1.31 | 0.011 |
| 3 | TC0100011477.hg.1 | joynybu | -1.05 | 0.022 | 74 | TC1600010849.hg.1 | ploserbo | 1.02 | 0.020 | 145 | TC1600010761.hg.1 | sheeseybo | 1.46 | 0.025 | 216 | TC0600006935.hg.1 | maflerby | 1.11 | 0.008 |
| 4 | TC2200008394.hg.1 | zykee | -1.53 | 0.010 | 75 | TC1700009886.hg.1 | ruveebu | 1.08 | 0.050 | 146 | TC1600011268.hg.1 | rasuru | 1.53 | 0.023 | 217 | TC0600008781.hg.1 | Y_RNA | 1.03 | 0.025 |
| 5 | TC0900008698.hg.1 | gortaby | -1.05 | 0.031 | 76 | TC0200014169.hg.1 | manemo | 1.03 | 0.035 | 147 | TC0200015877.hg.1 | verdee | 1.07 | 0.009 | 218 | TC0400006985.hg.1 | | 1.12 | 0.023 |
| 6 | TC0X00010532.hg.1 | AMOT; MIR4329 | -1.14 | 0.020 | 77 | TC1000010775.hg.1 | | 1.01 | 0.021 | 148 | TC1700007783.hg.1 | poywer | 1.04 | 0.005 | 219 | TC1700007420.hg.1 | storsmy | 1.08 | 0.014 |
| 7 | TC1000008465.hg.1 | NHP2P1 | -1.11 | 0.013 | 78 | TC0900008847.hg.1 | LCN2 | 3.89 | 0.030 | 149 | TC1600008324.hg.1 | teyzabu | 1.01 | 0.004 | 220 | TC0900009734.hg.1 | | 1.55 | 0.030 |
| 8 | TC0300009555.hg.1 | | -1.14 | 0.039 | 79 | TC1100006995.hg.1 | SAA1 | 2.42 | 0.031 | 150 | TC1900008499.hg.1 | gloywu | 1.09 | 0.005 | 221 | TC0X00010499.hg.1 | | 1.55 | 0.034 |
| 9 | TC0200014904.hg.1 | | -1.04 | 0.037 | 80 | TC1100013152.hg.1 | SAA2; SAA2-SAA4 | 2.78 | 0.032 | 151 | TC1900007009.hg.1 | weyflor | 1.02 | 0.036 | 222 | TC1100012123.hg.1 | MMP7 | 1.27 | 0.045 |
| 10 | TC2000006451.hg.1 | | -1.25 | 0.038 | 81 | TC0200014847.hg.1 | | 1.10 | 0.048 | 152 | TC0100012462.hg.1 | spuplar | 1.33 | 0.021 | 223 | TC0400011218.hg.1 | | 1.01 | 0.005 |
| 11 | TC0900007270.hg.1 | zoybarby | -1.10 | 0.015 | 82 | TC0500009766.hg.1 | CTC-338M12.9 | 1.07 | 0.049 | 153 | TC0100012792.hg.1 | plerlarbu | 1.34 | 0.030 | 224 | TC0100014185.hg.1 | dymubo | 1.21 | 0.005 |
| 12 | TC0600011267.hg.1 | AL121932.1 | -1.42 | 0.037 | 83 | TC0100007010.hg.1 | | 1.13 | 0.049 | 154 | TC0400011577.hg.1 | husume | 1.02 | 0.043 | 225 | TC0X00010498.hg.1 | smoyva | 1.09 | 0.008 |
| 13 | TC0200007134.hg.1 | SNORA64 | -1.47 | 0.024 | 84 | TC1000010782.hg.1 | saramu | 1.12 | 0.031 | 155 | TC1000010179.hg.1 | rawloy | 1.13 | 0.017 | 226 | TC0600013757.hg.1 | SOD2 | 1.57 | 0.001 |
| 14 | TC1100011545.hg.1 | ANAPC15 | -1.58 | 0.029 | 85 | TC0100008192.hg.1 | | 1.08 | 0.008 | 156 | TC1700009315.hg.1 | | 1.23 | 0.048 | 227 | TC1600009525.hg.1 | | 1.14 | 0.001 |
| 15 | TC2000009619.hg.1 | PPP4R1L | -1.02 | 0.017 | 86 | TC0700007952.hg.1 | storslerbu | 1.00 | 0.013 | 157 | TC1700010856.hg.1 | C1QL1 | 1.05 | 0.046 | 228 | TC1700006558.hg.1 | RP11-135N5.3 | 1.03 | 0.003 |
| 16 | TC0300008472.hg.1 | tisore | -1.08 | 0.027 | 87 | TC1300007795.hg.1 | | 1.11 | 0.006 | 158 | TC1000010185.hg.1 | | 1.08 | 0.012 | 229 | TC1700007213.hg.1 | RNU6-1057P | 1.18 | 0.001 |
| 17 | TC0500010743.hg.1 | | -1.32 | 0.011 | 88 | TC0300010335.hg.1 | tawderbo | 1.07 | 0.005 | 159 | TC0600009855.hg.1 | skoyjee | 1.18 | 0.010 | 230 | TC0900010918.hg.1 | parplawbu | 1.29 | 0.001 |
| 18 | TC0600013738.hg.1 | | -1.07 | 0.016 | 89 | TC1800008232.hg.1 | josaw | 1.43 | 0.007 | 160 | TC1000010181.hg.1 | jarnorby | 1.04 | 0.013 | 231 | TC0200012526.hg.1 | torpawbo | 1.15 | 0.000 |
| 19 | TC0700008077.hg.1 | SNORA14A | -2.04 | 0.009 | 90 | TC1000012132.hg.1 | | 1.01 | 0.007 | 161 | TC1800006892.hg.1 | luror | 1.01 | 0.020 | 232 | TC1800008329.hg.1 | | 1.09 | 0.000 |
| 20 | TC1100010216.hg.1 | PLEKHA7 | -1.64 | 0.033 | 91 | TC1900011419.hg.1 | | 1.08 | 0.007 | 162 | TC1200006731.hg.1 | shyblabu | 1.48 | 0.044 | 233 | TC0400011820.hg.1 | moklorby | 1.47 | 0.001 |
| 21 | TC0400010989.hg.1 | ADAMTS3 | -1.14 | 0.022 | 92 | TC1400007980.hg.1 | yokiru | 1.20 | 0.017 | 163 | TC1900009614.hg.1 | snawtubu | 1.23 | 0.021 | 234 | TC0500008831.hg.1 | sleyjy | 1.08 | 0.000 |
| 22 | TC0300013723.hg.1 | RNU6-42P | -1.01 | 0.010 | 93 | TC2200008688.hg.1 | dorzoy | 1.29 | 0.024 | 164 | TC0100009904.hg.1 | porplo | 1.01 | 0.036 | 235 | TC1900008023.hg.1 | varjer | 1.08 | 0.000 |
| 23 | TC1300008906.hg.1 | fawfarby | -1.16 | 0.003 | 94 | TC0300013124.hg.1 | parkubu | 1.43 | 0.003 | 165 | TC0100013012.hg.1 | | 1.25 | 0.019 | 236 | TC1600009537.hg.1 | dorwoy | 1.47 | 0.001 |
| 24 | TC0700008583.hg.1 | AP1S1 | -1.15 | 0.032 | 95 | TC0100012224.hg.1 | korero | 1.22 | 0.002 | 166 | TC1500007094.hg.1 | RP11-151N17.1 | 1.08 | 0.041 | 237 | TC2000008089.hg.1 | ZGPATandLIME1andS | 1.91 | 0.000 |
| 25 | TC0400011297.hg.1 | RNU6-33P | -1.06 | 0.016 | 96 | TC0900007014.hg.1 | RNU4ATAC11P | 1.35 | 0.002 | 167 | TC1500009454.hg.1 | gawpee | 1.12 | 0.020 | 238 | TC0200007375.hg.1 | | 1.12 | 0.011 |
| 26 | TC0400011366.hg.1 | RNU6-34P | -1.38 | 0.017 | 97 | TC0300008769.hg.1 | | 1.16 | 0.017 | 168 | TC0500010781.hg.1 | SLC38A9 | 1.16 | 0.038 | 239 | TC0700007861.hg.1 | snuslerbu | 1.23 | 0.025 |
| 27 | TC0200014994.hg.1 | RNU6-1; RNU6-9; R | -1.06 | 0.013 | 98 | TC1100012428.hg.1 | pkinase.69 | 1.03 | 0.022 | 169 | TC0X00007842.hg.1 | | 1.45 | 0.009 | 240 | TC0400010487.hg.1 | nybabo | 1.08 | 0.044 |
| 28 | TC0700012465.hg.1 | RNU6-1; RNU6-9; R | -1.06 | 0.013 | 99 | TC0100007845.hg.1 | MANEAL | 1.07 | 0.003 | 170 | TC1900009060.hg.1 | | 1.01 | 0.047 | 241 | TC1900007432.hg.1 | seypey | 1.02 | 0.049 |
| 29 | TC0X00010986.hg.1 | RNU6-1; RNU6-9; R | -1.06 | 0.013 | 100 | TC0300012378.hg.1 | charvawbo | 1.04 | 0.007 | 171 | TC1900008192.hg.1 | slorseybu | 1.19 | 0.017 | 242 | TC0200007454.hg.1 | spuboybu | 1.10 | 0.008 |
| 30 | TC1400008874.hg.1 | RNU6-8 | -1.06 | 0.013 | 101 | TC0600007897.hg.1 | blarspabu; flerjaw; p | 1.05 | 0.025 | 172 | TC2000006523.hg.1 | vuwy | 1.11 | 0.014 | 243 | TC0700009694.hg.1 | varshoby | 1.08 | 0.017 |
| 31 | TC1500009854.hg.1 | RNU6-1 | -1.06 | 0.013 | 102 | TC1000010612.hg.1 | | 1.06 | 0.023 | 173 | TC0300010507.hg.1 | RNU6-922P | 1.10 | 0.022 | 244 | TC0500006804.hg.1 | peyskoby | 1.04 | 0.003 |
| 32 | TC1700010689.hg.1 | kenema | -1.49 | 0.020 | 103 | TC1000010182.hg.1 | MIR938 | 1.09 | 0.024 | 174 | TC0100013075.hg.1 | ferpabu | 1.19 | 0.018 | 245 | TC0800008850.hg.1 | zarvoyby | 1.03 | 0.001 |
| 33 | TC0100017817.hg.1 | SNORA14B | -1.76 | 0.047 | 104 | TC0400007581.hg.1 | | 1.12 | 0.017 | 175 | TC0900007107.hg.1 | | 1.12 | 0.008 | 246 | TC1600008167.hg.1 | glawoy | 1.19 | 0.001 |
| 34 | TC0700012045.hg.1 | RNU6-1104P | -1.22 | 0.042 | 105 | TC1100009452.hg.1 | | 1.17 | 0.026 | 176 | TC0600009406.hg.1 | C6orf58 | 1.59 | 0.014 | 247 | TC1200012264.hg.1 | moswee | 1.08 | 0.002 |
| 35 | TC1800007597.hg.1 | RNU6-39P | -1.13 | 0.049 | 106 | TC1800009118.hg.1 | swarso | 1.13 | 0.047 | 177 | TC1700010546.hg.1 | smyklee | 1.05 | 0.004 | 248 | TC0400006566.hg.1 | teyglu | 1.02 | 0.035 |
| 36 | TC1900011438.hg.1 | RNU6-222P | -1.49 | 0.041 | 107 | TC0300013147.hg.1 | slukeebu | 1.17 | 0.004 | 178 | TC0X00008912.hg.1 | kuku | 1.15 | 0.043 | 249 | TC0100016728.hg.1 | | 1.54 | 0.034 |
| 37 | TC1400008860.hg.1 | | -1.17 | 0.049 | 108 | TC1100006885.hg.1 | sherflerbu | 1.09 | 0.007 | 179 | TC0Y00006888.hg.1 | soro | 1.15 | 0.043 | 250 | TC1700010951.hg.1 | snuslaw | 1.08 | 0.014 |
| 38 | TC0100010921.hg.1 | HMCN1 | -1.22 | 0.007 | 109 | TC1600006478.hg.1 | vuru | 1.02 | 0.005 | 180 | TC0300009505.hg.1 | floygobo | 1.06 | 0.005 | 251 | TC0700008050.hg.1 | STAG3L1andTRIM73a | 1.06 | 0.037 |
| 39 | TC0X00007295.hg.1 | SSXP5 | -1.23 | 0.005 | 110 | TC1100009822.hg.1 | flucher | 1.04 | 0.018 | 181 | TC0700012183.hg.1 | sparblee | 1.02 | 0.006 | 252 | TC1700006526.hg.1 | warseebo | 1.01 | 0.030 |
| 40 | TC0300011646.hg.1 | | -1.02 | 0.007 | 111 | TC1700009338.hg.1 | feesmee | 1.51 | 0.004 | 182 | TC1100010042.hg.1 | pleykerby | 1.17 | 0.011 | 253 | TC0200016136.hg.1 | | 1.15 | 0.009 |
| 41 | TC2000008364.hg.1 | | -1.16 | 0.004 | 112 | TC0700011907.hg.1 | zawkar | 1.10 | 0.010 | 183 | TC1100007012.hg.1 | RP11-504G3.2 | 1.01 | 0.004 | 254 | TC0200012744.hg.1 | blarklee | 1.17 | 0.008 |
| 42 | TC0100011597.hg.1 | LOC102723833; RP | -1.12 | 0.024 | 113 | TC1700009418.hg.1 | | 1.17 | 0.018 | 184 | TC1100007908.hg.1 | basha | 1.07 | 0.005 | 255 | TC1600009706.hg.1 | shoysmee | 1.43 | 0.006 |
| 43 | TC1100011766.hg.1 | | -1.31 | 0.018 | 114 | TC0700012157.hg.1 | rykaw | 1.31 | 0.009 | 185 | TC0200013608.hg.1 | kloyfa | 1.11 | 0.004 | 256 | TC0900007845.hg.1 | plawtawbo | 1.12 | 0.005 |
| 44 | TC1100008587.hg.1 | RP11-885L14.1 | -1.08 | 0.049 | 115 | TC1000007597.hg.1 | | 1.10 | 0.015 | 186 | TC1200012086.hg.1 | parney | 1.26 | 0.014 | 257 | TC1200008008.hg.1 | | 1.32 | 0.002 |
| 45 | TC0100007233.hg.1 | PPP1R11P1 | -1.24 | 0.018 | 116 | TC0400006460.hg.1 | | 1.01 | 0.013 | 187 | TC0100014157.hg.1 | slawmybo | 1.09 | 0.014 | 258 | TC1700006649.hg.1 | skyvarbu | 1.39 | 0.003 |
| 46 | TC0600007270.hg.1 | HIST1H2AC | -1.16 | 0.005 | 117 | TC1300008085.hg.1 | shupa | 1.10 | 0.009 | 188 | TC2200006444.hg.1 | LA16c-13E4.3 | 1.21 | 0.006 | 259 | TC1500010073.hg.1 | | 1.10 | 0.001 |
| 47 | TC1600007996.hg.1 | RPL23AP91 | -1.00 | 0.021 | 118 | TC0200016277.hg.1 | plujobu | 1.56 | 0.002 | 189 | TC1100007471.hg.1 | pluloby | 1.06 | 0.045 | 260 | TC1600007414.hg.1 | skypaw | 1.07 | 0.014 |
| 48 | TC1700009949.hg.1 | SREBF1 | -1.87 | 0.035 | 119 | TC1200011910.hg.1 | | 1.24 | 0.004 | 190 | TC0X00011187.hg.1 | blarnar | 1.14 | 0.037 | 261 | TC1200006473.hg.1 | skeeblarbu | 1.18 | 0.021 |
| 49 | TC1000007360.hg.1 | | -1.28 | 0.010 | 120 | TC1200011937.hg.1 | zeycho | 1.38 | 0.002 | 191 | TC1100007915.hg.1 | jeesha | 1.10 | 0.013 | 262 | TC1900006702.hg.1 | swerber | 1.05 | 0.021 |
| 50 | TC1800009047.hg.1 | tuflu | -1.24 | 0.043 | 121 | TC0400008059.hg.1 | bleebybo | 1.34 | 0.001 | 192 | TC0500012347.hg.1 | RNU7-156P | 1.06 | 0.033 | 263 | TC2200008787.hg.1 | kiyu | 1.13 | 0.022 |
| 51 | TC0500012964.hg.1 | mawka | -1.08 | 0.005 | 122 | TC1900007625.hg.1 | swutawbu | 1.25 | 0.003 | 193 | TC0600009832.hg.1 | snoU13 | 1.05 | 0.031 | 264 | TC1800007094.hg.1 | smapler | 1.03 | 0.034 |
| 52 | TC1200010559.hg.1 | VDR | -1.12 | 0.001 | 123 | TC1500009301.hg.1 | RP11-96O20.5 | 1.09 | 0.002 | 194 | TC0700007969.hg.1 | klosharby | 1.29 | 0.034 | 265 | TC0900007350.hg.1 | bleeskobu | 1.12 | 0.032 |
| 53 | TC2100007783.hg.1 | | -1.05 | 0.009 | 124 | TC0X00006804.hg.1 | | 1.15 | 0.002 | 195 | TC0700011009.hg.1 | slershaby | 1.04 | 0.026 | 266 | TC0900010078.hg.1 | fawvobu | 1.12 | 0.032 |
| 54 | TC0100009228.hg.1 | dermerbo | -1.13 | 0.023 | 125 | TC1100007694.hg.1 | flerny | 1.11 | 0.005 | 196 | TC0200014227.hg.1 | wydorbu | 1.17 | 0.025 | 267 | TC0200015315.hg.1 | slawstoyby | 1.05 | 0.046 |
| 55 | TC0100015204.hg.1 | cherpeebu | -1.20 | 0.000 | 126 | TC1900009933.hg.1 | C19orf42 | 1.35 | 0.006 | 197 | TC0500006837.hg.1 | ferslawbu | 1.31 | 0.025 | 268 | TC1700007789.hg.1 | | 1.07 | 0.027 |
| 56 | TC0100017861.hg.1 | beerawbo | -1.21 | 0.020 | 127 | TC1900009457.hg.1 | | 1.17 | 0.002 | 198 | TC0400011965.hg.1 | | 1.19 | 0.010 | 269 | TC1400010322.hg.1 | | 1.09 | 0.020 |
| 57 | TC0600007342.hg.1 | | -1.01 | 0.014 | 128 | TC1700010926.hg.1 | ninoma | 1.04 | 0.034 | 199 | TC1100010041.hg.1 | ST5 | 1.09 | 0.009 | 270 | TC1500008709.hg.1 | lyswee | 1.04 | 0.036 |
| 58 | TC1900010151.hg.1 | RP11-678G14.5 | -1.03 | 0.034 | 129 | TC0300013154.hg.1 | | 1.94 | 0.021 | 200 | TC0600013764.hg.1 | | 1.02 | 0.012 | 271 | TC0200010980.hg.1 | CCL20 | 4.20 | 0.045 |
| 59 | TC0200014974.hg.1 | nariro | -1.04 | 0.016 | 130 | TC0600006933.hg.1 | tureme | 1.10 | 0.040 | 201 | TC1100007907.hg.1 | termuby | 1.25 | 0.013 | 272 | TC1700007636.hg.1 | PIGW | 1.01 | 0.020 |
| 60 | TC1700010183.hg.1 | | -1.11 | 0.013 | 131 | TC0100006776.hg.1 | larnarbu | 1.02 | 0.024 | 202 | TC0900008852.hg.1 | rawvarby | 1.44 | 0.026 | 273 | TC0100017469.hg.1 | joybo | 1.18 | 0.033 |
| 61 | TC1200011661.hg.1 | | -1.04 | 0.019 | 132 | TC0100007329.hg.1 | | 1.00 | 0.043 | 203 | TC1700011455.hg.1 | tuwaw | 1.18 | 0.044 | 274 | TC2000009522.hg.1 | CYP24A1 | 1.66 | 0.036 |
| 62 | TC2200006464.hg.1 | muyora | -1.02 | 0.015 | 133 | TC1600008243.hg.1 | chersnee | 1.20 | 0.043 | 204 | TC0100018083.hg.1 | mimaha | 1.09 | 0.003 | 275 | TC0800006981.hg.1 | plersheyby | 1.05 | 0.043 |
| 63 | TC0600014276.hg.1 | HLA-DMB | -1.06 | 0.026 | 134 | TC2100008139.hg.1 | | 1.26 | 0.032 | 205 | TC1200007616.hg.1 | chawjorby | 1.02 | 0.005 | 276 | TC1000012183.hg.1 | | 1.22 | 0.047 |
| 64 | TC0700011796.hg.1 | SAMD9 | -1.02 | 0.032 | 135 | TC0100009364.hg.1 | CSF1 | 1.35 | 0.003 | 206 | TC0100011517.hg.1 | | 1.22 | 0.006 | 277 | TC1700007513.hg.1 | weeserbo | 1.16 | 0.042 |
| 65 | TC1300008501.hg.1 | TIMM8BP1 | -1.02 | 0.029 | 136 | TC0400011014.hg.1 | CXCL5 | 2.71 | 0.002 | 207 | TC1000007273.hg.1 | RNU7-77P | 1.19 | 0.001 | 278 | TC0600009851.hg.1 | MTHFD1L | 1.01 | 0.032 |
| 66 | TC0300013451.hg.1 | RP11-42D20.1 | -1.01 | 0.022 | 137 | TC1000008721.hg.1 | | 1.01 | 0.003 | 208 | TC1400010043.hg.1 | SERPINA6 | 1.04 | 0.002 | 279 | TC0X00006699.hg.1 | | 1.07 | 0.040 |
| 67 | TC1700012382.hg.1 | OMG | -1.02 | 0.011 | 138 | TC0600006929.hg.1 | peygleybu | 1.04 | 0.001 | 209 | TC1600010655.hg.1 | mawry | 1.19 | 0.001 | 280 | TC1100009172.hg.1 | zyfer | 1.04 | 0.029 |
| 68 | TC0100016442.hg.1 | | 1.54 | 0.042 | 139 | TC1000008720.hg.1 | pykleebu | 1.37 | 0.000 | 210 | TC0900010914.hg.1 | boyshee | 1.01 | 0.014 | 281 | TC1900009615.hg.1 | foysey | 1.04 | 0.014 |
| 69 | TC0100013500.hg.1 | steypeybo | 1.14 | 0.017 | 140 | TC1800008192.hg.1 | smeeree | 1.01 | 0.000 | 211 | TC0100009962.hg.1 | spawbee | 1.26 | 0.044 | 282 | TC1200009218.hg.1 | woytybo | 1.01 | 0.019 |
| 70 | TC0600009561.hg.1 | | 1.13 | 0.021 | 141 | TC0400006477.hg.1 | flawvarbo | 1.26 | 0.000 | 212 | TC1700012077.hg.1 | RP11-498C9.15 | 1.04 | 0.039 | 283 | TC1700009389.hg.1 | | 1.32 | 0.030 |
| 71 | TC0200010030.hg.1 | AC096649.2; plerly | 1.22 | 0.039 | 142 | TC1600007558.hg.1 | | 1.01 | 0.001 | 213 | TC1600009385.hg.1 | RP11-876N24.5 | 1.05 | 0.025 | 284 | TC2000009724.hg.1 | sukey | 1.02 | 0.020 |

**Figure S1. Hsu *et al.*, 2021**

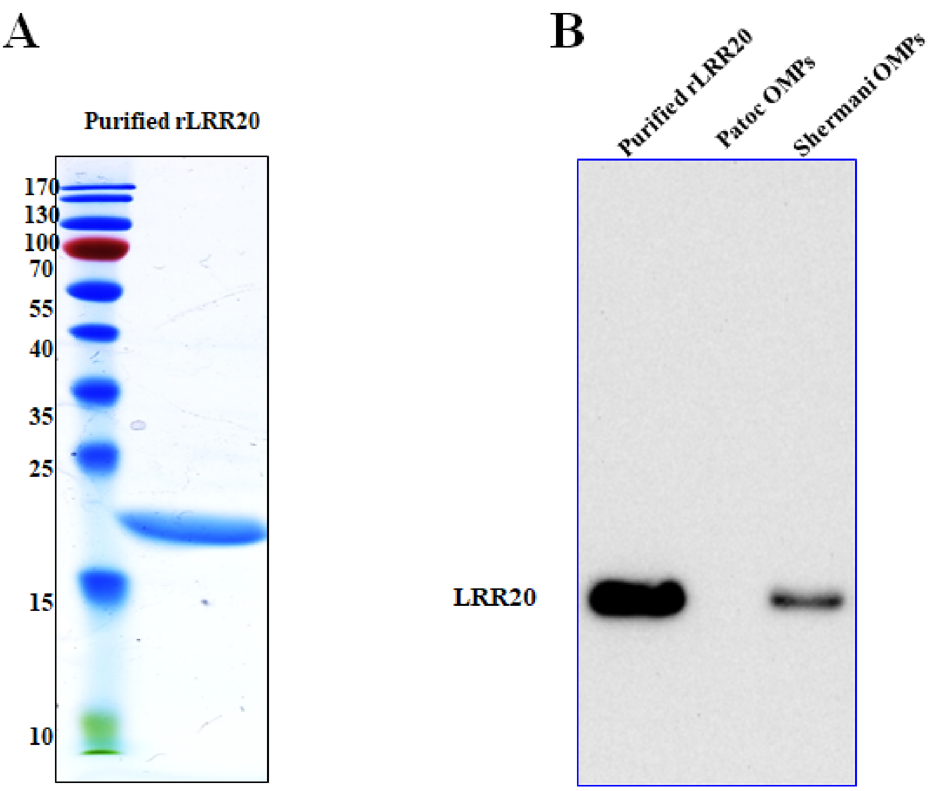

**Figure S2. Hsu *et al.*, 2021**

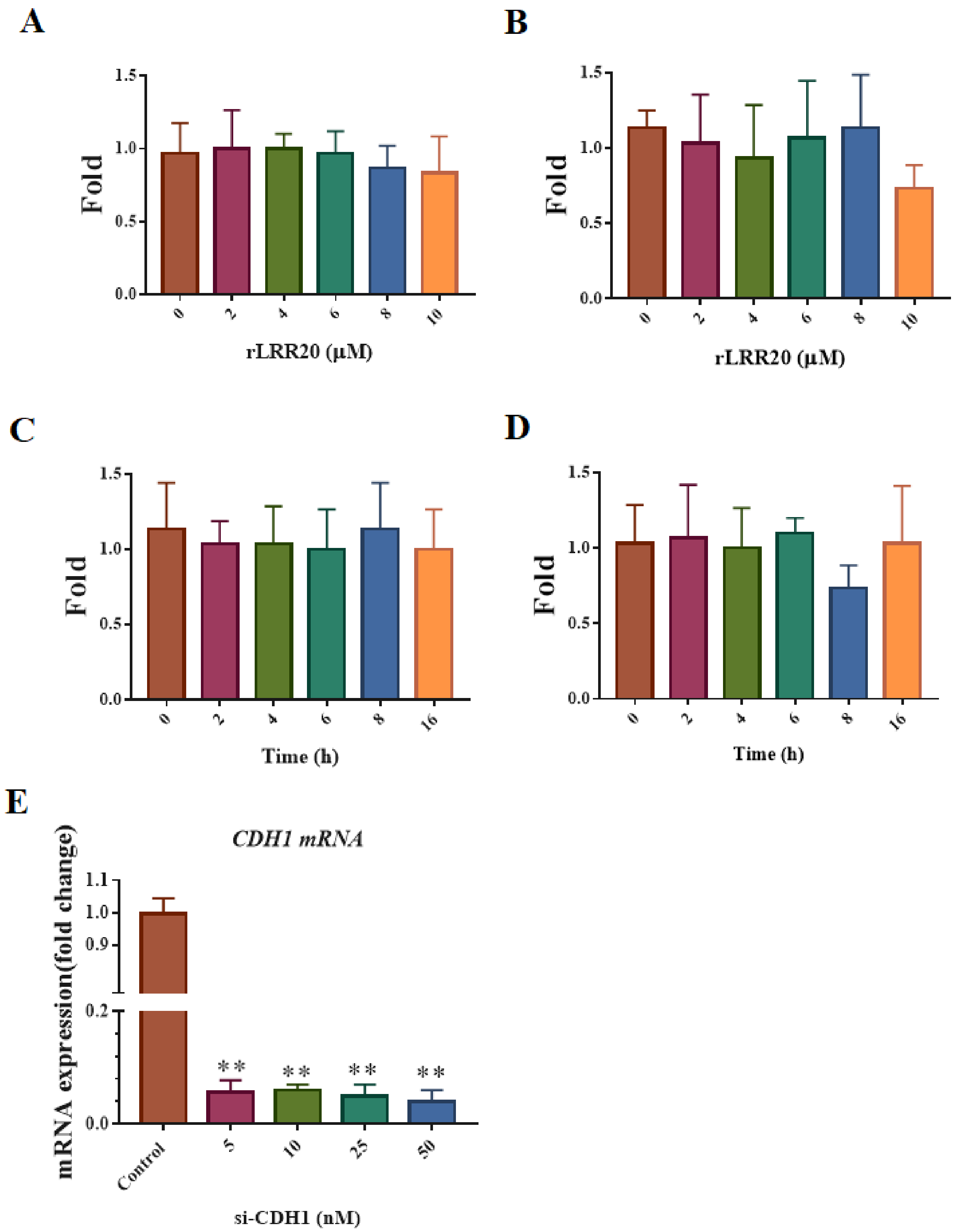

Figure S3. Hsu *et al.*, 2021

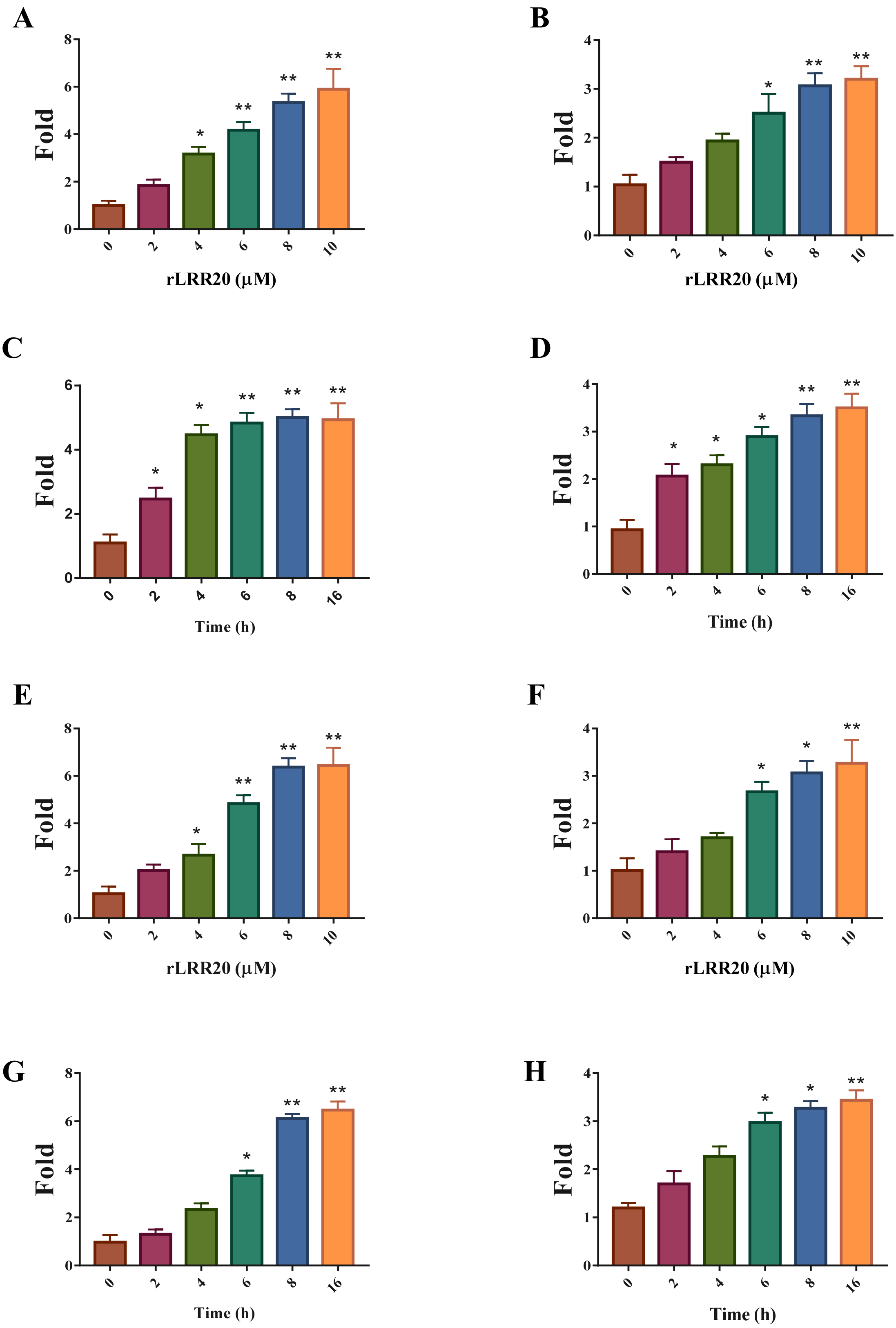

**Figure S4. Hsu *et al.*, 2021**

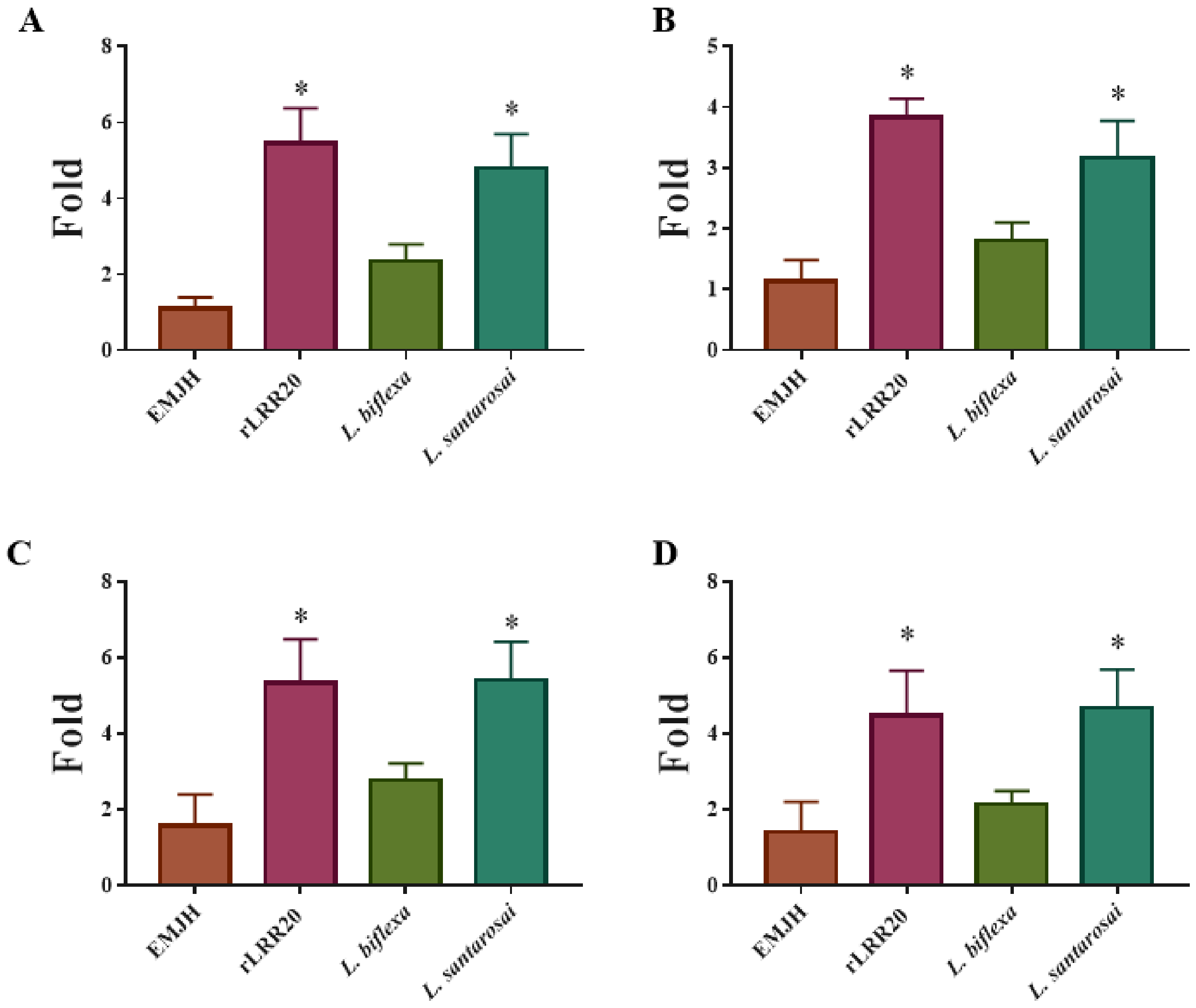

**Figure S5. Hsu *et al.*, 2021**

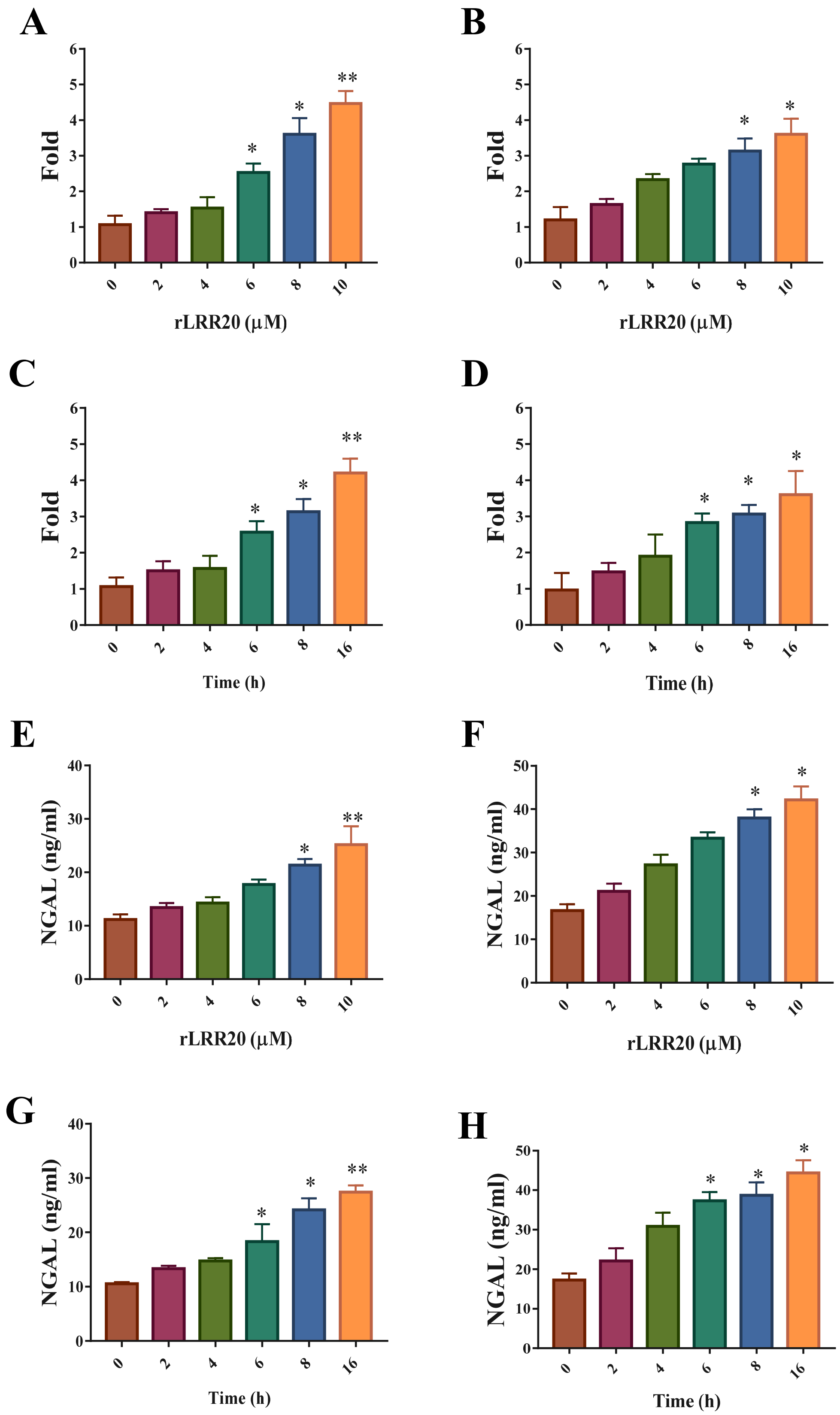

**Figure S6. Hsu *et al.*, 2021**

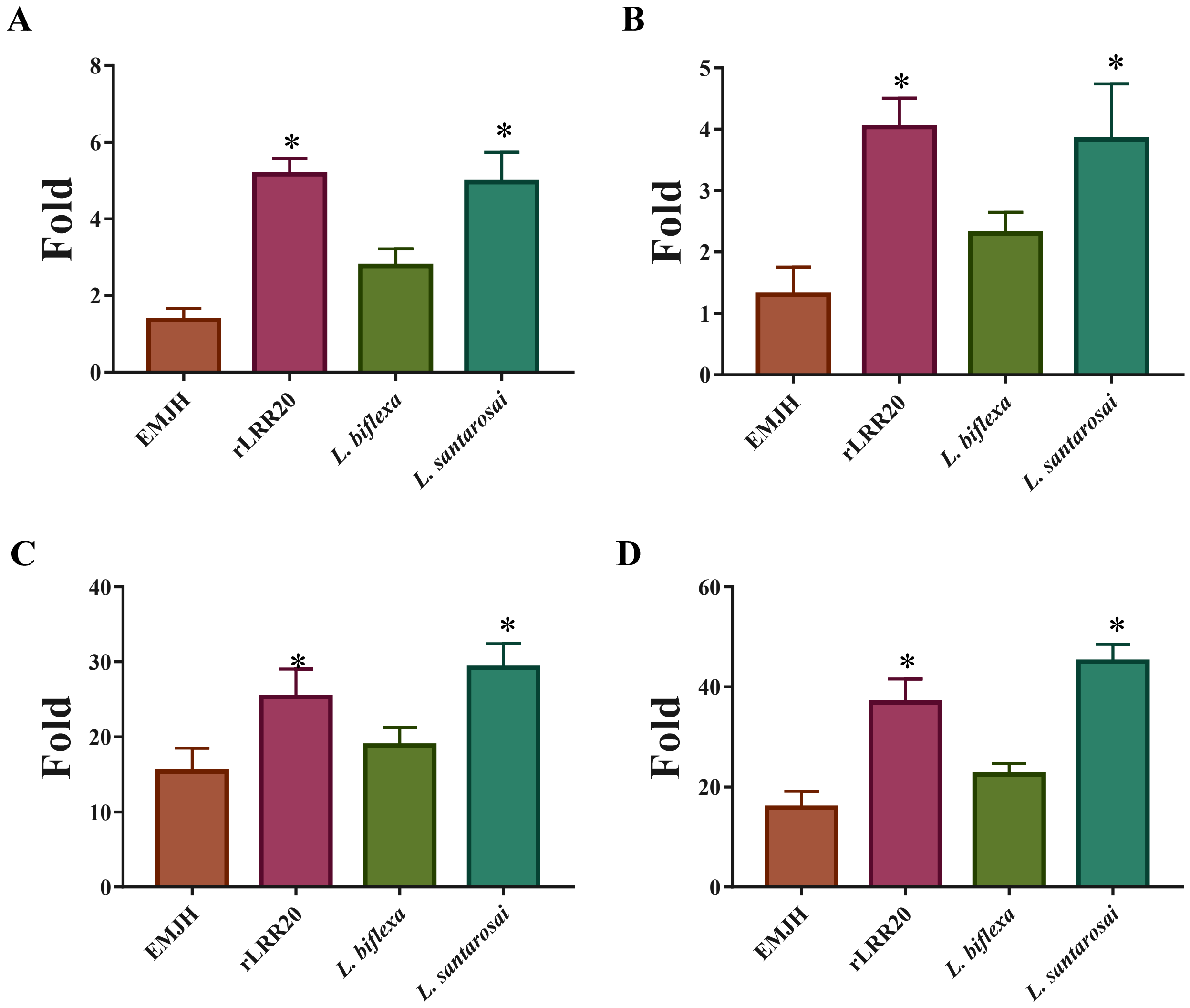

Supplement: Supplementary file 1 [file ijms-22-13132-s001.zip › ijms-1459232-supplementary.pdf]
